# Chaperonin-Containing TCP1 Complex (CCT) Promotes Breast Cancer Growth Through Correlations With Key Cell Cycle Regulators

**DOI:** 10.3389/fonc.2021.663877

**Published:** 2021-04-30

**Authors:** Heba Ghozlan, Adrian Showalter, Eunkyung Lee, Xiang Zhu, Annette R. Khaled

**Affiliations:** ^1^ Division of Cancer Research, Burnett School of Biomedical Sciences, College of Medicine, University of Central Florida, Orlando, FL, United States; ^2^ Department of Health Sciences, College of Health Professions and Sciences, University of Central Florida, Orlando, FL, United States

**Keywords:** tumorigenesis, luminal A, proliferation, node, targeting, oncogene

## Abstract

Uncontrolled proliferation as a result of dysregulated cell cycling is one of the hallmarks of cancer. Therapeutically targeting pathways that control the cell cycle would improve patient outcomes. However, the development of drug resistance and a limited number of inhibitors that target multiple cell cycle modulators are challenges that impede stopping the deregulated growth that leads to malignancy. To advance the discovery of new druggable targets for cell cycle inhibition, we investigated the role of Chaperonin-Containing TCP1 (CCT or TRiC) in breast cancer cells. CCT, a type II chaperonin, is a multi-subunit protein-folding complex that interacts with many oncoproteins and mutant tumor suppressors. CCT subunits are highly expressed in a number of cancers, including breast cancer. We found that expression of one of the CCT subunits, CCT2, inversely correlates with breast cancer patient survival and is subject to copy number alterations through genomic amplification. To investigate a role for CCT2 in the regulation of the cell cycle, we expressed an exogenous CCT2-FLAG construct in T47D and MCF7 luminal A breast cancer cells and examined cell proliferation under conditions of two-dimensional (2D) monolayer and three-dimensional (3D) spheroid cultures. Exogenous CCT2 increased the proliferation of cancer cells, resulting in larger and multiple spheroids as compared to control cells. CCT2-expressing cells were also able to undergo spheroid growth reversal, re-attaching, and resuming growth in 2D cultures. Such cells gained anchorage-independent growth. CCT2 expression in cells correlated with increased expression of MYC, especially in spheroid cultures, and other cell cycle regulators like CCND1 and CDK2, indicative of a novel activity that could contribute to the increase in cell growth. Statistically significant correlations between CCT2, MYC, and CCND1 were shown. Since CCT2 is located on chromosome 12q15, an amplicon frequently found in soft tissue cancers as well as breast cancer, CCT2 may have the basic characteristics of an oncogene. Our findings suggest that CCT2 could be an essential driver of cell division that may be a node through which pathways involving MYC, cyclin D1 and other proliferative factors could converge. Hence the therapeutic inhibition of CCT2 may have the potential to achieve multi-target inhibition, overcoming the limitations associated with single agent inhibitors.

## Introduction

Breast cancer is the most common cancer among women and a leading cause of death. Worldwide estimates of age-standardized incidence rate and mortality rate for breast cancer are 46.3 and 13.0 per 100,000, respectively (1). Breast cancer is typically classified based on the expression of estrogen receptor (ER), progesterone receptor (PR), and human epidermal growth factor receptor 2 (HER2). The main molecular subtypes of breast cancer are: luminal A, which is hormone-receptor positive (ER+PR+HER2-, Ki67 low), low grade, grows slowly and has the best prognosis; luminal B, which is also hormone-receptor positive (ER+PR+HER2+/-, Ki67 high), grows faster than luminal A and has a worse prognosis; HER2 positive or enriched, which is hormone receptor negative but HER2 positive (ER-PR-HER2+), grows faster than luminal cancers, and has a worse prognosis; and triple negative (TNBC) or basal, which is hormone receptor negative (ER-PR-HER2-), is more invasive, is common in women with BRCA1 mutations and cannot be treated with endocrine therapies or HER2 inhibitors as with the other subtypes. These subtypes of breast cancer help stratify patients and impact prognostic predictions and therapeutic decision making (2–4). However, breast cancer is a heterogeneous disease with complexities that go beyond these subtypes. Loss of tumor suppressors and amplification of oncogenes are common in breast tumors. Gene amplification is the most frequent genetic alteration in breast cancer with MYC, CCND1, epidermal growth factor receptor (EGFR), fibroblast growth factor receptor (FGFR), CDK4, and MDM2 being the genes most frequently amplified in primary and recurrent breast tumors (3, 5–10). Genetic alterations in these genes correlate with patient prognosis and clinicopathological features (11–13) and their therapeutic targeting remains the goal of precision medicine.

Genetic alterations support malignant transformations that results from uncontrolled proliferation, especially those that deregulate cell cycle phases – specifically the Gap 1 (G1) phase to Synthesis (S) phase transition (14). Therapeutically targeting cancer proliferative pathways is therefore of significant interest (15). The cell cycle is tightly regulated by cyclin dependent kinases (CDK), cyclins, and inhibitors such as p21 or p27 as well as proteolytic pathways to drive cell progression from the G1 phase through S phase, Gap 2 (G2) phase, then mitosis and cytokinesis. The G1 to S transition serves as a key checkpoint through regulation of the activities of retinoblastoma protein (Rb) and histone deacetylases, which control transcription through E2 factor (E2F). In breast cancer, as with other cancers, deregulation of the G1/S transition results in uncontrolled entry into S, bypassing the checkpoint (14, 16). Normally, mitotic signals drive the expression of cyclin D, which associates with the G1 kinases, CDK4/6, leading to the phosphorylation of Rb, activation of the transcriptional activity of E2F, and the subsequent generation of the S phase kinase, CDK2/cyclin E. In breast cancer, ER+ in particular, multiple signaling pathways converge to target cyclin D (17, 18). MYC is also involved in cell proliferation and differentiation, transcriptionally activating cell cycle regulators and repressing cell cycle inhibitors and is implicated in the development of cancer drug resistance (19–21). With that said, treatment options for breast cancer patients depend on the molecular classification of their tumors and the availability of targeted therapeutics. Recent successes with CDK inhibitors, such as those that target CDK4, are encouraging (22–24), since these inhibitors block the proliferation of cancer cells. However, there are no drugs in clinical use that target CCND1/cyclin D1 or MYC. Endocrine therapies remain the best option for ER+ patients (luminal cancers), but the development of drug resistance is problematic. Patients can also develop resistance to CDK4 inhibitors (25), three of which are approved for clinical use (26). Identifying new targetable oncogenes will advance knowledge on the evolution and progression of cancer and reveal novel treatment approaches that improve the prognosis and long-term survival of cancer patients, especially those that develop resistance to front-line therapies.

To advance the discovery of targetable factors for cancer therapy, we focused on CCT, which is a type II eukaryotic chaperonin that is composed of two stacked rings consisting of eight distinct subunits (CCT1-8) that form the protein folding chamber. Each CCT subunit assembles at a specific location in relation to other subunits in cis (same) and trans (opposite) positions in the ring. A CCT subunit contains three domains: an equatorial domain that forms the base of the chamber, an intermediate domain that has the ATP binding pocket, a hinge that attaches to the apical domain, and the apical domain itself. The apical domain has multiple hydrophobic areas that bind different substrates; hence substrate binding is not based on the amino acid sequence of a protein but rather its structural features. CCT subunits have different binding affinities to ATP, which has a regulatory role, and as well as different binding affinities for substrates (27, 28). While the scope and breadth of the CCT interactome are not fully understood, reports suggest that CCT could interact with about 1-15% of the proteome (29–31) to support cellular processes such as those involved in proliferation, cell cycle progression, and invasion. Cytoskeleton proteins, actin and tubulin, are obligate substrates for CCT (32). In addition, direct interactions with CCT and cell cycle proteins, transcription factors, and tumor suppressors like PLK1 (33), cdc20 (34, 35), CDH1 (36), Cyclin E (37), p53 (38), STAT3 (39) and others (30) are reported. Hence, cancer cells could become highly dependent on CCT to provide the functional, folded forms of many oncoproteins and essential factors required for survival and growth.

We and others reported that CCT subunits were highly expressed in breast cancer as compared to normal tissue and that their expression increased with patients’ tumor stage and metastasis (40, 41). Of the CCT subunits, we found that CCT2 expression inversely correlated with the overall survival of breast cancer patients (40, 42). CCT2 could thus be a novel oncogene and serve as a prognostic biomarker, which supports deeper investigation into the role of the chaperonin complex and, CCT2 in particular, in the process of carcinogenesis. Most of our understanding of the role of CCT2 in cancer is inferred from the activity of the whole CCT folding complex and identified protein-protein interactions. To augment this data, we specifically examined the role of CCT2 in cell cycle progression using 2D and 3D cultures to investigate cellular and molecular changes directly associated with overexpressing the CCT2 subunit in luminal A cells, the most common subtype of breast cancer. We found that CCT2 expression drove the proliferation of cancer cells in spheroid cultures as well as in 2D monolayers, endowing cancer cells with growth adaptivity irrespective of anchorage. CCT2 expression also correlated with increased expression of key proliferative factors, such as MYC, especially in spheroid cultures, suggesting that the chaperonin could have a role regulating the expression of these oncogenes. These findings present CCT2 as a potential cell cycle regulator and possible proto-oncogene with prognostic and therapeutic value in breast and other cancers. 

## Materials and Methods

### Cell Lines and Generation of CCT2 Overexpressing or Depleted Cells

Cell lines used were MCF7 (ATCC HTB-22) human ER+ breast cancer cells, T47D (ATCC HTB-133) human ER+ breast cancer cells, and E0771 (CH3 Biosystems) murine TNBC cells. T47D cells were cultured in RPMI-1640 (Corning) supplemented with 10% fetal bovine sera (FBS) (Gemini), 1% penicillin-streptomycin (P/S) (Corning), and 0.2 units/mL human recombinant insulin (Santa Cruz). MCF7 cells were cultured in Eagle’s Minimum Essential Medium (EMEM) (ATCC) supplemented with 10% FBS (Gemini), 1% P/S (Corning), and 0.01 mg/mL human recombinant insulin (Santa Cruz). MCF7 and T47D cells were transduced with plasmids for the lentiviral control or CCT2-FLAG as previously described (40). For selection, cells were maintained with 0.5 μg/mL puromycin dihydrochloride (ThermoFisher) and microscopically observed for GFP expression. E0771 TNBC (CH3 Biosystems) were cultured in RPMI-1640 (Corning) supplemented with 10% FBS (Gemini) and 1% P/S (Corning). E0771 were transduced with lentiviral-based inducible small hairpin RNA (shRNA) to deplete CCT2 as previously reported (40). To induce shRNA expression, 0.5 µg/mL doxycycline was added to the media for 24-72 hours.

### Spheroid Formation Assay

Cells were grown in ultra-low attachment plate (ULA) (Corning) and supplemented with complete growth media as appropriate for each cell line. To observe the formation of multiple spheroids, 24-well flat bottom ULA plates were seeded with 30,000 cells/well. To observe the formation of individual spheroids, 96-well rounded bottom ULA plates were seeded with 1,000 cells/well. Culture day 0 refers to the start of spheroid plating. Spheroids were grown for 8 days on ULA plates and assayed at different time points: days 3, 5, and 8. Brightfield images and overlay GFP images were captured using the Cytation 5 Cell Imaging Multi-Mode Reader (BioTek).

### 2D Post 3D Cultures (Spheroid Growth Reversal)

To assess spheroid growth reversal, day 8 spheroids grown in 24-well ULA plates were collected and disintegrated either physically by repeat pipetting or chemically using Accumax (Innovative Cell Technology), washed once in PBS, resuspended in complete media, and then plated in standard tissue culture T-25 flask with media appropriate to each cell line as follows. To assess anchorage independent growth, spheroids were chemically dissociated using Accumax and plated onto standard tissue culture flasks. Brightfield images were acquired at endpoints 2-3 weeks post transfer at 20X magnification (AXIO Observer). Alternatively, intact day 8 spheroids from 96-well ULA plates were transferred to 48-well standard tissue culture plates with media appropriate to each cell line. Fluorescent images were captured using the Cytation 5 (BioTek), and brightfield microscopy images were captured using the AXIO Observer with the 20X objective (Carl Zeiss AG) at days 2 and 5 post-transfer.

### Proliferation and Viability Assays

The ViaFluor^®^ 405 SE Cell Proliferation Kit (Biotium) was used to assess proliferation by tracking the number of cell divisions. Cells from day 3 spheroid cultures were stained following manufacturer’s protocol, incubated for 48 hours, then collected on day 5 for analysis. As a reference population, a subset of cells from day 3 spheroids were stained and immediately collected for analysis. Samples were analyzed by flow cytometry using the Cytoflex S flow cytometer (Beckman Coulter). The viability of cells was assessed using propidium iodide (PI) (Invitrogen) staining in an exclusion assay. Five µl of (1mg/ml) of PI were added to 200 µl cell suspension of 10^6^ cell/ml concentration. Samples were analyzed by flow cytometry (Cytoflex S). Data analysis was performed using FCS Express 6 software (DeNovo).

### Cell Cycle Analysis

To assess cell cycle progression, 200,000 cells/well were added to 6-well tissue culture plates and supplemented with complete growth media. Cells were cultured overnight. To induce growth arrest, cells were washed and then supplemented with serum-free media and cultured for 24 hours. Initiation of growth was synchronized by the addition of complete growth media with 10% FBS and cells assessed after 24- and 48-hours. Samples were collected for PI intracellular staining as follows. Briefly, equal volumes of detergent buffer and PI (Invitrogen) solution were added to cells at 10^6^ cells/ml followed by the addition of 15 µl RNAase solution (Thermo Scientific) per 1ml total volume. Cells were incubated for 3 hours at room temperature. Samples were analyzed by flow cytometry (Cytoflex S). Data analysis was performed using FCS Express 6 software (DeNovo).

Detergent buffer recipe: 8 gm sodium chloride, 0.4 gm potassium chloride, 0.06 gm KH_2_PO_4_, 0.09 gm Na_2_HPO_4_, 0.14 gm CaCl_2_, 0.10 gm MgCl_2_, 0.10 gm MgSO_4_, 5.6 gm HEPES, 2 gm bovine serum albumin (BSA), 4 gm Nonidet P-40 in 1000 ml distilled water.

PI staining solution recipe: 25 gm PI in 500 ml detergent buffer.

RNAase solution recipe: 0.006 g RNAase in 1 ml distilled water.

### Adhesion Assay

Spheroids were grown on 96 well ULA plates as described above for 8 days. On day 8, the spheroids were transferred to 96-well standard tissue culture plates (Eppendorf) and allowed to attach for 3 hours. After 3 hours, spheroids were washed to remove remaining floating cells and imaged using the Cytation 5 multi-model plate reader (BioTek). The following day, the spheroids were imaged again with the Cytation 5 and then lifted from the plate. Spheroids were then dissociated using ice-cold Accumax (Innovative Cell Technologies) with shaking for 15 min. Once dissociated, the cells were counted by imaging using Cytation 5 reader and Gen 5 software based on green fluorescent protein (GFP) levels. 

### Immunofluorescence Staining for Confocal Microscopy

Spheroids grown on 24-well ULA plates were collected and chemically dissociated using Accumax (Innovative Cell Technologies). Dissociated spheroid cells were plated on poly-L-lysine coated 25 mm coverslips (Fisher Scientific), and cells were left for 1 hour to adhere before fixation. Cells were fixed with 4% PFA for 10 min, washed with PBS for 10 min, permeabilized in 0.5% TritonX-100 in PBS for 5 min, blocked with 1% BSA and 0.05% Tween in PBS for 1 hour, stained with and ActinRed™ 555 ReadyProbes™ Reagent (Rhodamine phalloidin) (Thermofisher) for 1 hour, and mounted with anti-fade DAPI (Invitrogen). Images were acquired with a Zeiss LSM 710 confocal microscope (Carl Zeiss AG) with the 40X objective. Total fluorescence per cell was analyzed using ImageJ. The RGB image was converted to a grayscale 8-bit image type, the region of interest was selected (each cell), and then area, mean and integrated intensity were measured using the Analyze tool in ImageJ. Corrected fluorescent intensity (CTCF) for each cell was calculated according to the equation: CTCF = Integrated Density – (Area of selected cell X Mean fluorescence of background readings). Each field had at least 6 images of cells at 40X magnification.

### Western Blot

Cell lysis, sodium dodecyl sulfate polyacrylamide gel electrophoresis (SDS-PAGE), total protein staining, gel visualization, and gel band quantification were performed as previously described (43). Lysates were collected from cells grown in 2D culture flasks or 24-well ULA plates. Total protein concentration was determined using the Pierce BCA Protein Assay Kit (Thermo Scientific) following the manufacturer’s protocol. Anti-CCT2 (ab109184) and anti-FLAG (ab1162) antibodies were obtained from Abcam, anti-CCT3 (MA5-27872) antibodies were from Invitrogen, and anti-CCT-beta (MAB10050) antibodies were from Millipore. Note that anti-CCT2 (Abcam) and anti-CCT-beta (Millipore) antibodies target the N-terminal amino acids 1-100 and C-terminal amino acids of human CCT-beta, respectively. Secondary antibodies used were IRDye 800CW and IRDye 680CW (LI-COR).

### Quantitative Real Time Polymerase Chain Reaction (RT-qPCR)

Total RNA was extracted from cells using Trizol (Ambion) following the manufacturer’s instructions. RNA concentration was determined using the NanoDrop instrument (Nanodrop 8000, ThermoFisher). Approximately 1 µg of RNA was reverse transcribed to cDNA using iScript reverse transcription Supermix for RT-qPCR (Bio-Rad) according to the manufacturer’s instruction. cDNA was diluted to 10 ng/µl. For RT-qPCR, a 20 µl PCR reaction was performed using 5 µl of Fast Syber Green Mastermix (Applied Biosystem), 0.2 forward primer, 0.2 reverse primer, and 2 µl cDNA (10 ng/ml) and RNase free water. PCR reactions were performed in duplicate. GAPDH was used as a reference gene. PCR reactions were performed using the Applied Biosystems QuantStudio 7 Flex Real-Time PCR system of 40 cycles of 95°C for 3 seconds and 62°C for 30 seconds. The melting curve was evaluated for each reaction to verify a single amplification product. Relative mRNA expression was calculated using 2^–ΔCt^ and fold change using 2^–ΔΔCt^ equations. Primers used are shown in **Table 1**.

**Table 1:** Primer pairs used in RT-qPCR assay.

CDK4	Forward: TCT GGT ACC GAG CTC CCG AA
	Reverse: GAT TTG CCC AAC TGG TCG G
CDK2	Forward: ATG GAG AAC TTC CAA AAG GTG GA
	Reverse: CAG GCG GAT TTT CTT AAG CG
Cyclin D1	Forward: GCG TCC ATG CGG AAG ATC
	Reverse: ATG GCC AGC GGG AAG AC
Cyclin E1	Forward: GCC AGC CTT GGG ACA ATA ATG
	Reverse: AGT TTG GGT AAA CCC GGT CAT
MYC	Forward: GGA GGC TAT TCT GCC CAT TTG
	Reverse: GAG GCT GCT GGT TTT CCA CTA
P27	Forward: ACC TGC AAC CGA CGA TTC T
	Reverse: CAG GCT TCT TGG GCG TCT G
P21	Forward: TGA GCC GCG ACT GTG ATG
	Reverse: GTC TCG GTG ACA AAG TCG AAG TT
GAPDH	Forward: GAA GGT GAA GGT CGG AGT CAA C
	Reverse: TGG AAG ATG GTG ATG GGA TTT C
CCT3	Forward: TCAGTCGGTGGTCATCTTTGG
	Reverse: CCTCCAGGTATCTTTTCCACTCT
CCT2-FLAG	Forward: CAG AGG TGA TTC TGC GTG TG
	Reverse: TGT CGT CGT CGT CCT TGT AG
CCT2	Forward: GTT GGA GAG AAG CCA CGA AG
	Reverse: GTT GCC AGA GCC TTT CAG TC

### Bioinformatics Analysis

Interrogation of The Cancer Genome Atlas (TCGA) database was accomplished using the websource cBioPortal for Cancer Genomics [http://cbioportal.org (44, 45)] to visualize copy number alteration, mutual exclusivity, and co-expression of genes in different studies. TCGA data were analyzed, and graphics were downloaded using the webtool. CCT subunits alterations were analyzed using the Catalog of Somatic Mutations in Cancer (COSMIC) (46). mRNA expression and copy number data from The Cancer Cell Line Encyclopedia (CCLE) databases were downloaded using the Xena browser (46, 47).

### Statistical Analysis

For statistical analysis of protein levels, imaging data, spheroid growth, adhesion, viability and proliferation data, Prism 8 (GraphPad) was used to determine statistical significance. Different groups were tested using Student’s t-test or ANOVA as relevant. P-values of less than 0.05 were considered statistically significant. For analysis of gene expression data, we first checked the raw Ct data for batch effect. If it existed, we then removed the batch effect using R package (Limma) prior to downstream gene expression analysis. After batch effect was corrected, we used the 2^-ΔΔCt^ method to calculate and normalize gene expression for each target gene, in which -ΔΔCt was calculated using the formula: -ΔΔCt=average(ΔCt control sample)-ΔCt treated sample, where ΔCt=Ct target gene – Ct housekeeping gene. In all subsequent analysis, we log2-transformed the fold change (2^-ΔΔCt^), i.e., used -ΔΔCt, to evaluate the effect of different treatments on gene expression of each target gene. Multiple mixed effect linear regression model was used to evaluate expression of each gene in response to the effects of cell line, treatment and time. Time was set as a continuous variable with value of 0 for day 0, 1 for day 3, 2 for day 5 and 3 for day 8. Multi-factor ANOVA analysis was performed to evaluate the effect of individual factors, such as cell line, overexpression of CCT2 and culture type, depending on expression of each target gene. Spearman correlation coefficient was applied to test the gene interaction among MYC, CCND1 and total CCT2. These statistical analyses were performed using Stata MP 15 (StataCorp LLC, 2019) and R packages. All tests were two-tailed with a significance level of α (type I error) <0.05. The p-values were adjusted based on Holm adjustment when multiple tests were conducted.

## Results

### Co-Occurrence of CCT2 Genetic Alterations With Cell Cycle Gene Alterations Is Suggestive of Functional Relationships

**Figure 1:**
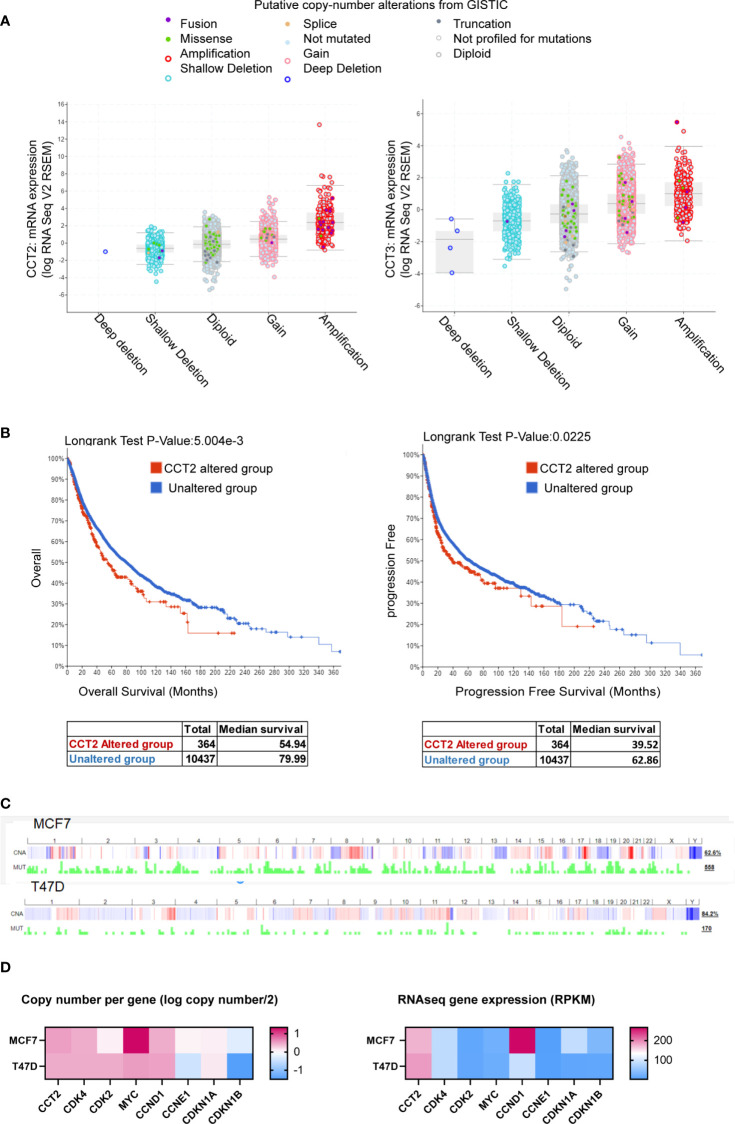
CCT2 is genomically amplified in cancer and correlates with reduced patient survival. **(A, B)** The TCGA PanCancer database was evaluated using cBioPortal for CCT2 and CCT3 mRNA expression and genetic alterations **(A)** and overall and progression free survival of patients **(B)** in CCT2 altered and unaltered groups. **(C)** Copy number alterations and mutation profiles for MCF7 and T47D cell lines based on CCLE data exported using cBioPortal are shown. **(D)** CCT copy number and expression in T47D and MCF7 cells, data were downloaded from The Cancer Cell Line Encyclopedia (CCLE) databases using the Xena browser. CCT subunit alterations in MCF7 and T47D were obtained from the COSMIC database and shown in **Table 3**.

The co-occurrence of genetic mutations that are functionally related contributes to the process of carcinogenesis. Identifying such patterns could reveal novel cancer initiating pathways and treatment targets. Genetic alterations in cell cycle regulators are common in breast cancer, supporting the uncontrolled proliferation of cancer cells. However, the heterogeneity of tumor types and subtypes, suggests that there is not a single mechanism that exerts a biological function like proliferation but rather different signaling pathways converge that are responsible for the complex dynamics of cancer growth. Identifying possible points of convergence for future therapeutic targeting underlies our studies. To this end, we mined pan-cancer databases like TCGA for the expression of CCT subunits in all cancers and showed that the most common type of copy number alteration of the CCT2 subunit gene was amplification, and that this gene was rarely deleted (**Figure 1A**). In comparison, data for CCT3 is included (**Figure 1A**). The importance of CCT2 in cancer progression is further emphasized by the fact that cancer patients with genetic alterations in CCT2 had reduced overall and progression free survival (**Figure 1B**). In support, we previously reported that breast cancer patients with CCT2 genetic alterations died up 70 months sooner than patients without alterations (40). These findings support investigating the relationship between CCT2 and cell cycle gene expression to reveal possible new pathways for therapeutic intervention. Since our previous studies showed that the CCT2 subunit was essential for breast cancer growth and tumor formation (40), we first examined whether alterations of the CCT2 gene associated with cell cycle gene alterations. As shown in **Table 2**, genetic alterations in CCT2 co-occurred with CDK4, CDK2, CCND1, and MYC.

**Table 2:** Co-occurring genetic alterations of CCT2 with cell cycle genes.

A	B	p-Value	Tendency
**CCT2**	**CDK4**	<0.001	Co-occurrence
MYC	CCNE1	<0.001	Co-occurrence
CCNE1	CDKN1A	<0.001	Co-occurrence
CCND1	CDK4	<0.001	Co-occurrence
**CCT2**	**CCND1**	<0.001	Co-occurrence
CDKN1A	CDKN1B	<0.001	Co-occurrence
CDK4	CDKN1A	<0.001	Co-occurrence
CDK4	CDKN1B	<0.001	Co-occurrence
CCND1	CDK2	<0.001	Co-occurrence
MYC	CDKN1A	<0.001	Co-occurrence
CDK4	CDK2	<0.001	Co-occurrence
CDK2	CDKN1B	0.001	Co-occurrence
**CCT2**	**MYC**	0.002	Co-occurrence
**CCT2**	**CDK2**	0.002	Co-occurrence
MYC	CDKN1B	0.002	Co-occurrence
MYC	CDK4	0.004	Co-occurrence
MYC	CCND1	0.005	Co-occurrence
CCNE1	CDKN1B	0.005	Co-occurrence

[From combined breast cancer studies (9103 patients/9524 samples in 17 studies) The Cancer Genome Atlas (TCGA)]. CCT2 gene interactions are highlighted in bold type.

As the platform to investigate a role for CCT2 in cell cycle regulation, we chose to use the T47D and MCF-7 cell lines. Both cell lines are luminal A epithelial breast cancer cells, ER+, and are derived from a metastatic site of pleural effusion. We previously reported that T47D and MCF7 cells had lower levels of cytosolic CCT2 protein as compared to basal cell lines like MDA-MB-231 (40, 42). Luminal A subtypes are among the most common breast cancer subtypes but also display molecular heterogeneity. Clinically this is reflected by differential treatment outcomes and the development of acquired endocrine therapy resistance, such as due to the overexpression/amplification of CCND1 and CDK4 (48, 49). The Cancer Cell Line Encyclopedia (CCLE) database for global and selected gene alterations indicates that MCF7 and T47D cell lines have variable copy number alteration profiles and mutation rates (**Figure 1C**). Focusing on the copy number and expression level of cell cycle genes, MCF7 cells have a relatively higher copy number of CCT2 and MYC but lower gene expression relative to T47D cells (**Figure 1D**). CCT2 gene expression is thus higher in T47D cells. MCF7 cells also had about the same copy number but relatively higher mRNA expression for CCND1 and CDKN1A compared to T47D cells (**Figure 1D**). Further, we examined whether these cell lines harbored any genetic alterations in CCT subunits. Using the COSMIC web-tool, we found that MCF7 and T47D cells had mutations in CCT6B and CCT8 subunits, respectively. In addition, CCT3 was overexpressed in MCF7 cells, but not in T47D cells (**Table 3**). These data indicate that, while T47D and MCF7 cell lines are representative of the most common forms of breast cancer, these display genetic heterogeneity that is observed in cancerous cells and thus could help reveal the role that CCT2 plays in the regulation of cancer cell growth, especially as cancers spread from local to disseminated disease. 

**Table 3:** Genetic alterations of CCT subunits in MCF7 and T47D cells.

Cell Line	CCT2 subunits alterations	
	Mutation	CNV and expression
**MCF7**	CCT6B (Substitution – intronic)	CCT3 is overexpressed
**T47D**	CCT8 (Substitution – intronic)	No changes reported

(COSMIC database).

### CCT2 Overexpression in Breast Cancer Cells Promotes the Growth of Spheroids

**Figure 2:**
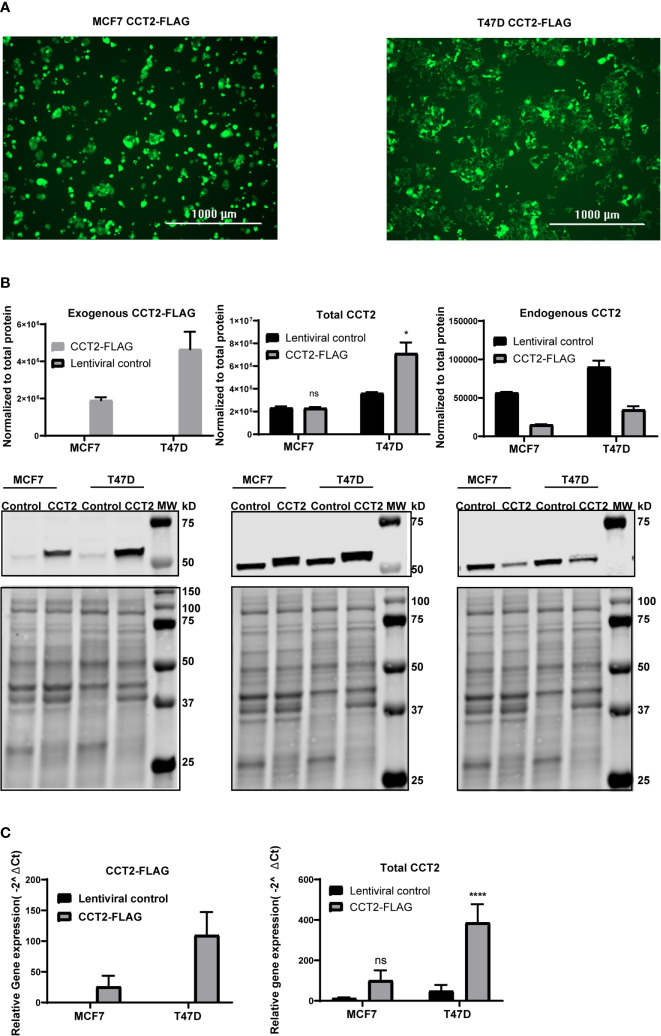
CCT2-FLAG is overexpressed in T47D and MCF7 breast cancer cell line. **(A)** Expression of GFP by cells transduced with lentiviral CCT2-FLAG and control vectors is shown. Images were acquired using the Cytation 5 Cell Imaging Multi-Mode Reader (BioTek). **(B)** Western blots for exogenous CCT2-FLAG (anti-FLAG antibody), total CCT2 (N-terminal specific anti-CCT2 antibody), and endogenous CCT2 (C-terminal specific anti-CCT2 antibody) proteins are shown. Data were normalized to total protein as described in *Methods*. Representative blots are shown (n=3), and data replicates summarized in the graph. **(C)** Relative mRNA expression for total CCT2 and exogenous CCT2-FLAG was determined by RT-qPCR (n=5). GAPDH was used as a reference gene. Calculations were based on using the equation 2^-ΔΔ Ct^ equation. Values are mean ± SD. *p-value <0.05, ****p-value <0.00005. ns, non-significant.

To achieve the exogenous expression of CCT2 in T47D and MCF7 cells, we used a lentiviral system to transduce cells with a FLAG-tagged CCT2 construct as described in our previous studies (40). As shown in **Figure 2A**, the transduction of both cell lines was equivalent as indicated by comparable levels of GFP expression from the lentiviral plasmid. CCT2-FLAG overexpression was examined by western blot using antibodies specific for the exogenous CCT2-FLAG protein (anti-FLAG), total CCT2 protein (anti-CCT2 N-terminal specific), and endogenous CCT2 protein (anti-CCT2 C-terminal specific). While both cell lines expressed the CCT2-FLAG protein, T47D cells had increased total CCT2 protein as compared to MCF7 cells (**Figure 2B**). This is in part due to the downregulation of endogenous CCT2 in CCT2-FLAG expressing cells (**Figure 2B**). In T47D cells, CCT2-FLAG expression increased total CCT2 mRNA (**Figure 2C**), which correlated with CCT2 protein expression. In MCF7 cells, CCT2-FLAG mRNA was increased as well; however, this did not correlate with increased total CCT2 protein as compared to lentiviral control, likely due to differences in post-transcriptional mechanisms. This difference in total CCT2 expression between the cell lines is important as we consider the effects of CCT2-FLAG overexpression upon cell growth, proliferation, and correlation with cell cycle regulators.

Since we observed that overexpressing CCT2-FLAG caused a decrease in endogenous CCT2, we determined whether other endogenous CCT subunits were also decreased. In MCF7 and T47D cells no statistically significant differences in endogenous CCT3 protein and mRNA expression were noted between CCT2-FLAG overexpressing and lentiviral controls (**Figure S1**). We previously reported that the endogenous levels of the other CCT subunits (e.g., CCT4, CCT5) were the same or increased in cells expressing CCT2-FLAG (40). Hence, the decrease in endogenous CCT2 upon expression of CCT2-FLAG was not accompanied by a similar decrease of other CCT subunits. Moreover, in our previous study we found that a major portion of the CCT2-FLAG protein physically associated with the other CCT subunits to form a complex (40). While this is suggestive that the biological activity of CCT2-FLAG in T47D and MCF7 cells is mediated as part of the CCT protein-folding oligomeric complex, it does not rule a novel possible biological function for the monomeric CCT2 mRNA or protein.

**Figure 3:**
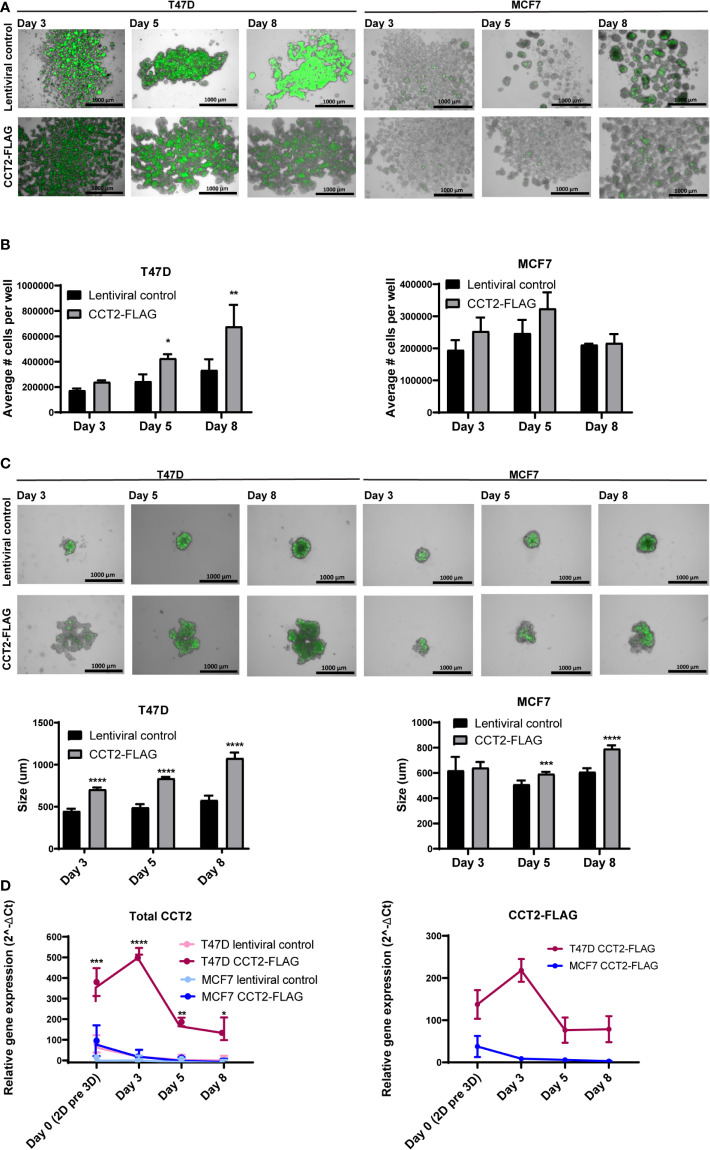
CCT2 enhances the formation of spheroids by breast cancer cells. **(A, B)** Merged brightfield and GFP images from T47D and MCF7 spheroids grown on 24-well ULA flat bottom plates at days 3, 5, and 8 of 3D culture are shown. Magnification was 2.5X. **(B)** Total spheroid cell count per well for T47D and MCF7 cells is shown (n=3). Cells were chemically dissociated from spheroids using Accumax and counted using flow cytometry. **(C)** Merged brightfield and GFP images of T47D and MCF7 spheroids grown on 96-well ULA round bottom plates at days 3, 5, and 8 of spheroid growth. Magnification was 2.5X. Single spheroids from 96-well ULA plates were used for perimeter measurements based on GFP signal using Gen5 software (n=5) is shown. **(D)** Relative mRNA expression for total CCT2 and exogenous CCT2-FLAG at day 0, day 3, day 5, day 8 (n=3-5) was determined by RT-qPCR. GAPDH was used as reference gene. Values are mean ± SD. *p-value <0.05, **p-value <0.005, ***p-value < 0.0005, ****p-value <0.00005.

3D culture models better mimic aspects of *in vivo* tumor growth dynamics compared to 2D cultures (50) and are a valuable tool to investigate tumor growth mechanisms. Cells grown in 3D culture form spheroids containing cores that are usually hypoxic and necrotic, having less access to growth factors, middle layers containing quiescent cells, and outer layers typically composed of actively proliferating cells in contact with the surrounding media. Utilizing 3D culture conditions, enabled through the use of ULA plates, we investigated the effect that CCT2-FLAG overexpression would have on spheroid formation by breast cancer cells. In flat bottom 24 well ULA plates, T47D and MCF7 cells expressing CCT2-FLAG formed multiple spheroids that spread across the plate as compared to cells expressing a lentiviral control plasmid (**Figure 3A**). Morphologically, the spheroids formed by T47D cells expressing CCT2-FLAG attained a sheet-like pattern rather than growing in small clusters of spheroids as seen with MCF7 cells expressing CCT2-FLAG or the T47D and MCF7 lentiviral controls (**Figure 3A**). Using the spheroids grown in 24 well ULA plates, we determined spheroid number per well at different days of spheroid formation (days 3, 5, and 8). A statistically significant increase in the number of cells forming spheroids was seen with T47D cells that overexpressed CCT2-FLAG, and a similar, though not statistically significant trend, was observed with MCF7 CCT2-FLAG expressing cells (**Figure 3B**). To measure the size of individual spheroids (by imaging single spheroids), we grew T47D and MCF7 CCT2-FLAG overexpressing and lentiviral control cells in 96-well rounded bottom ULA plates. For T47D and MCF7 CCT2 overexpressing cells, larger spheroids formed that increased at each time point - 3, 5 and 8 days (**Figure 3C**). We confirmed that CCT2-FLAG protein levels were maintained in each cell line through day 5 of spheroid growth, while in lentiviral control cells, endogenous CCT2 protein was downregulated (**Figures S2**, **S3**). T47D CCT2-FLAG over-expressing cells maintained higher total CCT2 mRNA that peaked at day 3 of the 3D culture (**Figure 3D**). These results demonstrate that increased expression of CCT2 can drive spheroid growth, even when normal cellular processes are decreasing, and that T47D cells, which maintain a higher expression level of total CCT2 than MCF7 cells, display corresponding increased growth of multiple and larger spheroids under 3D culture conditions.

**Figure 4:**
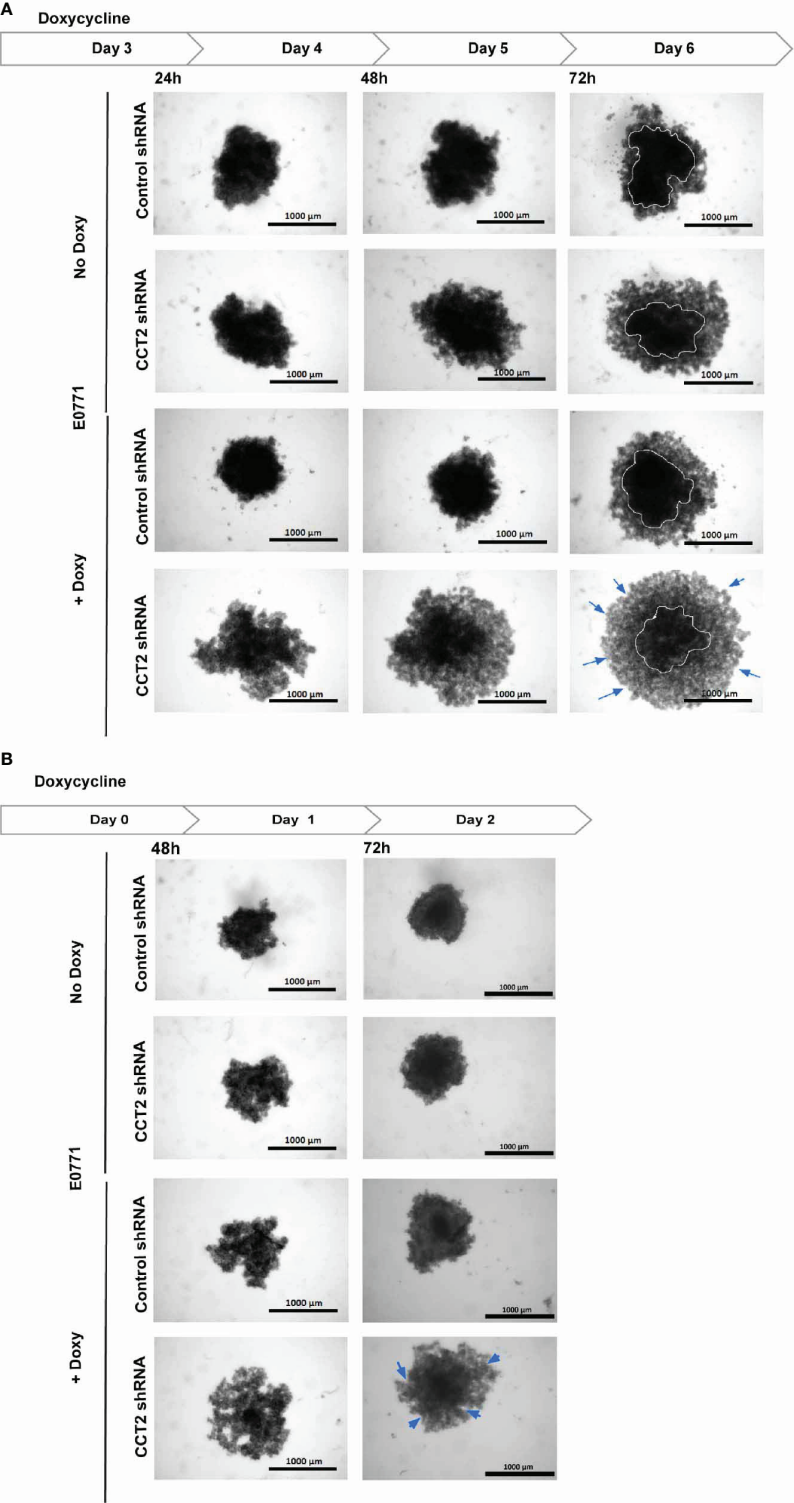
CCT2 depletion impairs breast cancer spheroid formation. **(A)** Brightfield images for E0771 cells at 24, 48, 72 hours after CCT2 depletion using 0.5 μg/ml doxycycline to induce CCT2 or control shRNA expression are shown. Cells were grown in round bottom 96-well ULA plates to form single spheroid in each well. Images were acquired using Cytation 5 Cell Imaging Multi-Mode Reader (BioTek) (n=5). Magnification was 2.5X. CCT2 depletion was induced at day 3 spheroid of spheroid growth. **(B)** CCT2 depletion in E0771 cells was induced at day 0 (start of spheroid cultures) and imaged at 48 hours and 72 hours after doxycycline was used to induce expression of CCT2 or control shRNA (n=5). Circles delineate morphology of dense cell aggregates, while arrows indicate the formation of loose cell aggregates.

To verify that CCT2 is essential for spheroid formation and growth, we performed a CCT2 depletion experiment using a doxycycline (doxy) inducible shRNA. A complete knockdown of CCT2 is not possible because CCT2 is an essential gene (depmap.org), and its loss causes death of cancer cells (40, 42, 43). Hence, we chose an inducible shRNA approach to achieve partial depletion. Previously, we established that 25-50% depletion of CCT2 caused loss of viability in MDA-MB-231 breast cancer cells and could inhibit tumor growth in a syngeneic mouse model of E0771 TNBC cells (40). We used these TNBC cells for CCT2 depletion because, unlike the luminal A breast cancer cells, these cells tend to express increased amounts of endogenous CCT2 and are amenable to depletion (40, 42). Using this same system to deplete CCT2, we plated the E0771 inducible control shRNA or CCT2 shRNA cells on 96-well ULA round bottom plates and assessed the growth of individual spheroids upon treatment with doxycycline (doxy) as follows. As shown in **Figure 4A**, E0771 cells were plated at day 0, and depletion of CCT2 was induced with doxy after day 3 of spheroid growth. Cells were imaged at 24-, 48-, and 72-hours post-doxy treatment. CCT2 depletion interfered with the formation of spheroids as these cells lost their tight interactions and loosely aggregated at the bottom of the well (**Figure 4A**); hence no measurements of spheroid size were acquired since mainly cell aggregates remained in the bottom of the wells of the ULA plates. As shown in **Figure 4B**, the experiment was repeated but instead CCT2 was depleted at the start of spheroid culture. When we induced CCT2 depletion on day 0, this prevented cells from forming tight spheroid structures and were loosely aggregated (**Figure 4B**). In both experiments, the resulting aggregates formed after CCT2 depletion were easily dispersed by pipetting, indicating that the tight cell-to-cell interactions needed to form spheroids were lost. These findings support that CCT2 promotes spheroid formation.

### CCT2 Overexpression Supports the Transition of Cells From 3D Spheroid to 2D Monolayer Cultures

**Figure 5:**
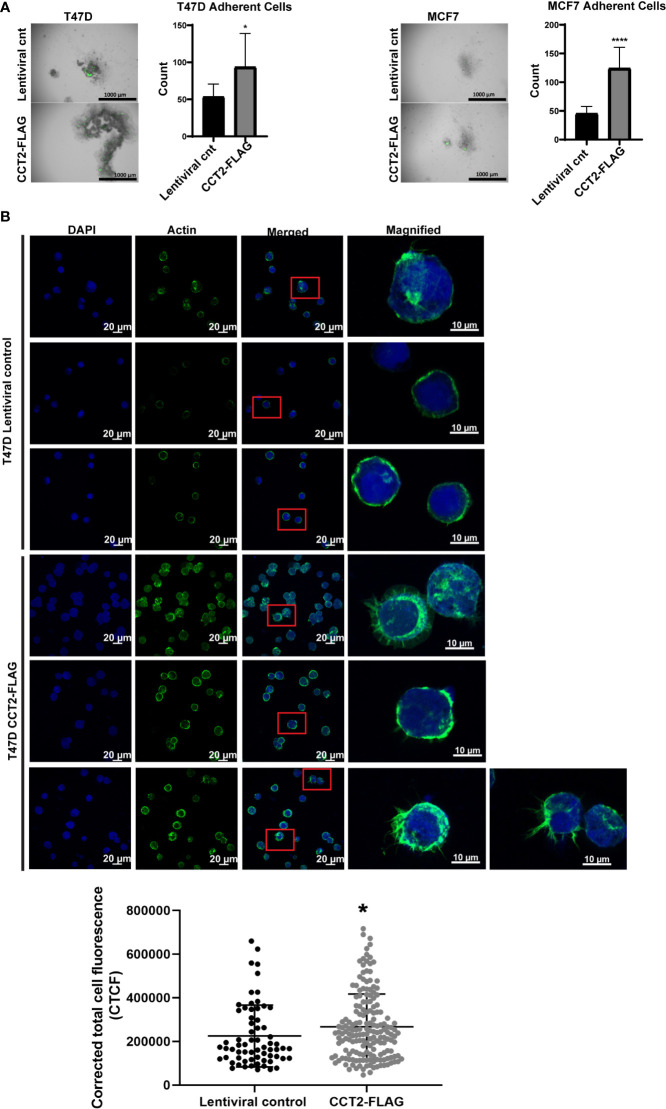
CCT2 overexpression promotes adherence of breast cancer cells in post-3D spheroid cultures and increases intracellular actin. **(A)** Brightfield and GFP overlay images of day 8 spheroids from T47D and MCF7 cells, CCT2-FLAG overexpressing and lentiviral control, transferred onto standard tissue culture plates are shown. After transfer, non-adherent cells were washed off and images were taken of remaining adherent cells. Adherent cells were then dissociated and counted by flow cytometry. Images were acquired using the Cytation 5 Cell Imaging Multi-Mode Reader (BioTek) (n=10). Magnification was 2.5X. **(B)** Confocal microscopy images of F-actin (stained with rhodamine phalloidin), DAPI, and overlays are shown for T47D, CCT2-FLAG overexpressing and lentiviral control, cells. Magnification was 40X. Inset shows a magnified view of cellular protrusions. To quantitate, integrated density was measured using ImageJ to calculate Corrected Total Cell Fluorescence (CTCF) as described in *Methods*. Values are mean with SD. *p-value <0.05, ****p-value <0.00005.

CCT2-FLAG overexpressing cells growing in 3D culture mimic aspects of *in vivo* tumors. We next wanted to determine whether these cells could undergo a reversal of 3D growth, re-attaching and expanding in a 2D monolayer. This model could mimic metastasis by retaining the malignant characteristics (e.g., invasiveness, stemness) of spheroid cells when these transition to 2D culture (51). Spheroids from day 8 cultures of T47D and MCF7 cells, lentiviral control and CCT2-FLAG expressing, were collected and transferred to standard tissue culture plates and allowed to re-attach for 3 hours. Cells were washed to remove unattached cells and then imaged and quantitated. More CCT2-FLAG expressing cells were able to re-attach as compared to lentiviral controls, suggesting that these cells preserved features that enabled re-establishment of growth in adherent cultures (**Figure 5A**). This was most apparent with T47D CCT2-FLAG expressing cells, since these retained cell-to-cell attachments, while also anchoring to a surface. Since actin is an obligate substrate of the CCT complex and has a role in spheroid formation by supporting cell-to-cell and cell-to-substrate interactions along with other cytoskeleton components (52), we microscopically examined the intracellular distribution of F-actin. T47D cells were chosen for F-actin visualization, since CCT2-FLAG overexpressing cells from this cell line displayed reversal of spheroid growth and increased re-attachment in monolayer culture (**Figure 5A**). We assessed F-actin levels by confocal microscopy using fluorescent phalloidin, which binds F-actin. CCT2-FLAG overexpression in T47D spheroid cells increased F-actin levels as compared to lentiviral control cells (**Figure 5B**). Interestingly, these cells also had more filopodial-like cellular protrusions compared to lentiviral control (**Figure 5B**). While MCF7 CCT2-FLAG overexpressing cells also underwent spheroid growth reversal (**Figure 5A**), in contrast to T47D cells, these cells did not attach strongly to coated coverslips and only a few cells could be imaged for F-actin by microscopy (data not shown). Collectively, these results show that CCT2 overexpression supports spheroid growth reversal and increased F-actin.

**Figure 6:**
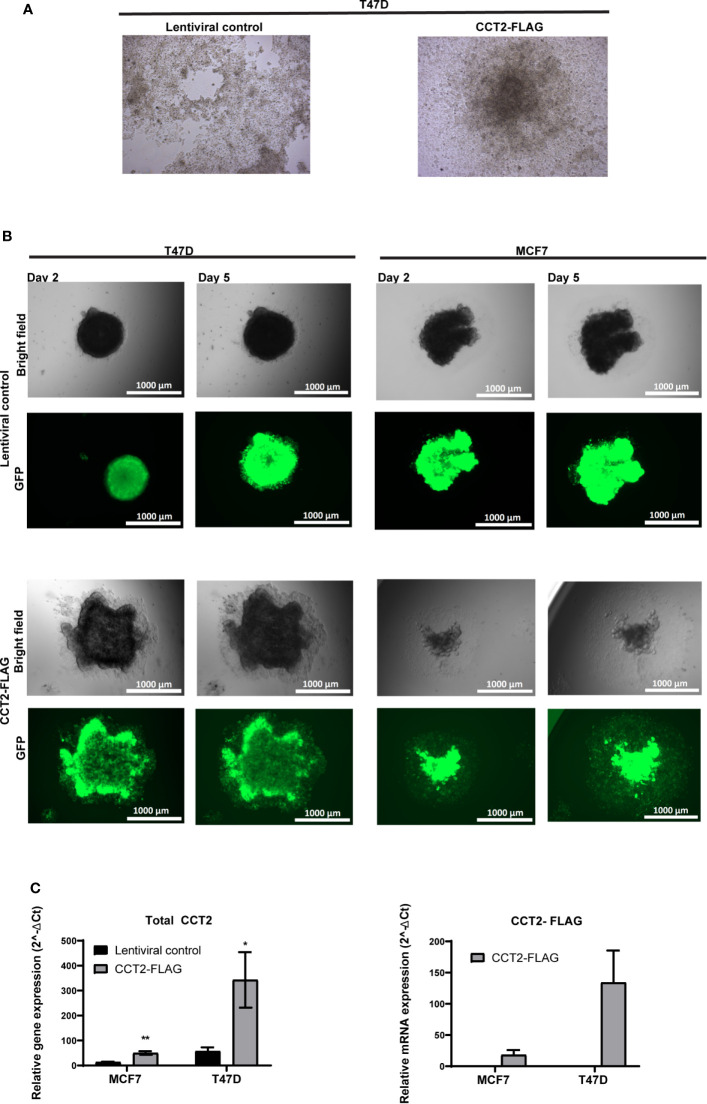
CCT2 supports transition of breast cancer cells from 3D to 2D monolayer culture. **(A)** Brightfield microscopic images are shown of 2D monolayer cultures transferred from spheroid derived T47D cells, CCT2-FLAG overexpressing and lentiviral control. Spheroids were chemically dissociated using Accumax and then plated into standard tissue culture flasks. Images were acquired 3 weeks post transfer (n=3). Magnification was 20X. **(B)** Brightfield and GFP microscopic images show results of spheroid transfer to standard tissue culture plates at days 2 and 5 post-transfer (n=5). Magnification was 2.5X. **(C)** Total CCT2 and CCT2-FLAG mRNA expression was evaluated from T47D and MCF7 cells, CCT2-FLAG overexpressing and lentiviral control, grown in 2D post-3D monolayer cultures (n=3) by RT-qPCR. The equation used was 2^-ΔΔ Ct^ and GAPDH was the reference gene as previously described. Values are mean with SD. *p-value <0.05, **p-value <0.005.

To further investigate the effect of CCT2-FLAG expression on spheroid growth reversal, we examined spheroids from T47D cells, CCT2-FLAG overexpressing and lentiviral control, under two experimental conditions for re-growth in 2D monolayers. First, we chemically disassociated day 8 spheroids and placed them in standard tissue culture plates for three weeks. Under these conditions, CCT2-FLAG overexpressing cells gained anchorage-independent growth as compared to lentiviral controls whose monolayer growth was limited by the dimensions of the well. As shown in **Figure 6A**, CCT2-FLAG overexpressing cells, in 2D culture conditions, grew independent of contact to the plate surface, forming a 3D spheroid-like structure that attached to the well. In a supplemental figure, we show additional images that demonstrate the transition of dissociated cells to spheroid-like structures in standard tissue culture plates (**Figure S4**). In the second approach, we transferred intact day 8 spheroids (not dissociated) from T47D and MCF7, CCT2-FLAG overexpressing and lentiviral control, cells to a standard tissue culture plate and imaged at different time points. CCT2-FLAG overexpression enhanced the transition of spheroids from 3D to 2D culture; by day 2 after the transfer, spheroids from CCT2-FLAG overexpressing cells were attached and growing faster than the lentiviral controls, especially the T47D cells (**Figure 6B**). These cells maintained CCT2-FLAG protein levels (**Figures S3**, **S5**) and higher total CCT2 mRNA (especially T47D cells) (**Figure 6C**). Based on these observations, we concluded that CCT2 overexpression confers to tumor cells enhanced growth potential and adaptability, even as these cells transition between suspension and adherent culture conditions, suggestive of the potential for invasive and metastatic-like behavior.

### CCT2 Overexpression Increases Cell Cycling in 3D and Post-3D Cultures

**Figure 7:**
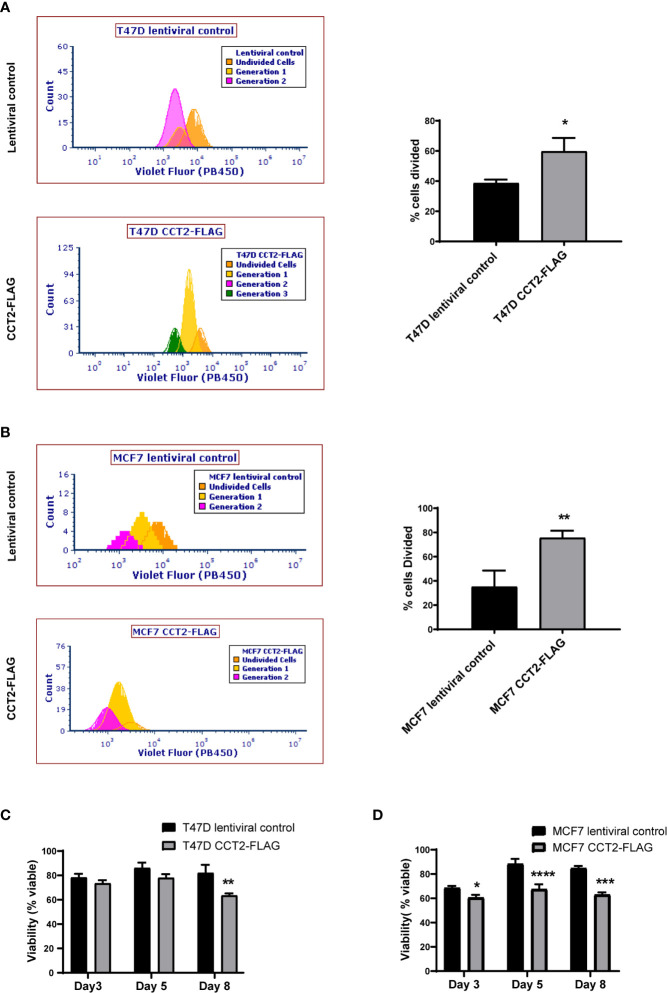
CCT2 overexpression increases cell division of breast cancer cells in 3D cultures. **(A, B)** The ViaFluor^®^ dye was used to assess cell division over time for T47D cells **(A)** and MCF7 cells **(B)**, CCT2-FLAG overexpressing and lentiviral controls, from days 3-5 of spheroid 3D cultures are shown. Cells were stained at day 3 and incubated for 48 hours before assessing cell division by flow cytometry. Generation time (histograms) and percent cells divided (graph) was determined (n=3). **(C, D)** PI exclusion assay was used to assess the viability of cells from spheroid cultures at days 3, 5, and 8 of 3D growth for T47D cells **(C)** and MCF7 cells **(D)** (n=3). Data was acquired using a CytoFlex S flow cytometer and analyzed using FCS Express software. Values are mean with SD. *p-value <0.05, **p- value <0.005, ***p-value <0.0005, ****p-value < 0.00005.

Having shown that overexpression of CCT2-FLAG resulted in larger spheroids and promoted spheroid growth reversal, we examined the proliferation of cells in 2D and 3D culture conditions using a dilution dye, ViaFluor^®^ 405. Cells in spheroid cultures (growing on ULA plates) were treated with the dye on day 3 and collected for analysis on day 5 of spheroid culture. The percent of cells divided during this period was significantly higher for the T47D and MCF7 cells overexpressing CCT2-FLAG as compared to lentiviral control. For T47D cells, 60% of CCT2-FLAG overexpressing cells divided over time compared with 40% of lentiviral control cells (**Figure 7A**). For MCF7 cells, about 80% of CCT2-FLAG overexpressing cells divided compared to about 40% of lentiviral control cells (**Figure 7B**). The viability of these cells was assessed on days 3, 5 and 8 using PI exclusion. Spheroids formed with CCT2-FLAG overexpressing cells had lower viability in later culture days compared to lentiviral control in both cell lines, which was most evident for MCF7 CCT2-FLAG overexpressing cells at days 5 and 8 of spheroid growth (**Figures 7C, D**; gating shown in **Figure S6**). Loss of viability in the T47D CCT2-FLAG overexpressing cells at day 8 could be explained by a bigger necrotic core in these larger spheroids (**Figure 3C**). Viability loss in MCF7 CCT2-FLAG overexpressing cells could be due to increased proliferation of cells that failed to thrive in spheroid cultures. In total, these findings provide additional support for CCT2 in promoting the proliferation of cancer cells and growth of spheroids.

**Figure 8:**
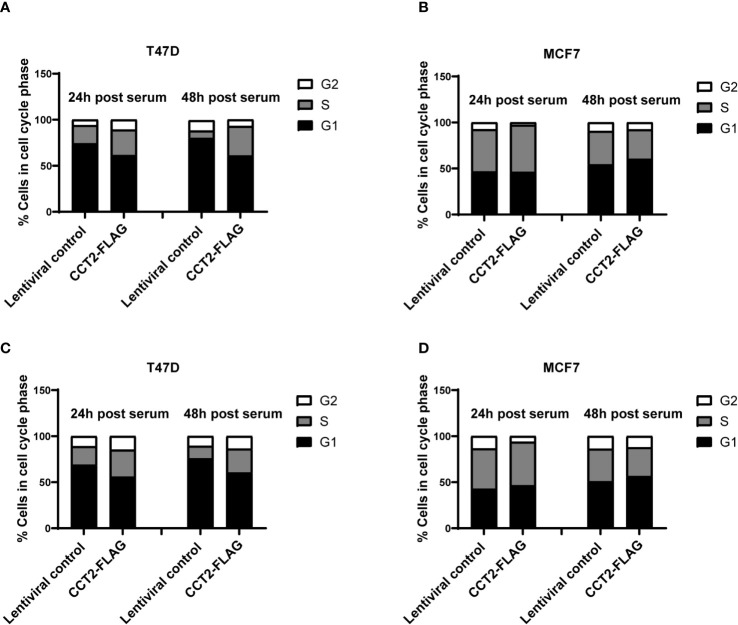
CCT2 overexpression promotes progression of breast cancer cells through the G1/S transition. Proliferation of T47D and MCF7 cells, CCT2-FLAG overexpressing and lentiviral control, in 2D cultures was synchronized after serum deprivation for 24 hours and cell cycle distribution was analyzed by PI intracellular staining of cells from 2D monolayer cultures **(A, B)** and 2D post 3D cultures **(C, D)** after 24 and 48 hours of serum introduction (n=3). Note that ~70% of the cells were synchronized in G1 after serum deprivation (**Figure S7**). Data was acquired using the CytoFlex S flow cytometer and analyzed with FCS Express software.

Based on our data mining showing the co-occurrence of CCT2 with cell cycle genes (**Table 2**) and our previous finding that overexpression of CCT2-FLAG promoted cell division in 2D monolayer cultures (40), we tested whether CCT2-FLAG overexpression could enhance cell cycle entry and progression. Cultures of T47D and MCF7, CCT2 overexpressing and lentiviral control, cells were synchronized for growth. After 24 hours of serum deprivation, cell cycle analysis using PI to stain the DNA confirmed that a majority of cells (~70%) were in G1 phase arrest (**Figures S7A–D**). Serum-containing media was introduced to the culture to synchronize growth, and cell cycling was assessed at 24- and 48-hours post-serum addition. Results showed that overexpression of CCT2-FLAG in T47D cells promoted the transition of cells from G1 to S and G2 phases of cell cycle. T47D cells overexpressing CCT2-FLAG had more cells in S and G2 phases at 24- and 48-hours post-serum addition compared to lentiviral control cells (**Figures 8A**, **S8A**). MCF7 CCT2-FLAG overexpressing and lentiviral control cells had about the same cell cycle distribution post-serum addition (**Figures 8B**, **S8B**).

We also established 2D monolayer cultures from the 3D cultures that underwent spheroid growth reversal – referred to as 2D post 3D. Using these cells, we tested whether CCT2-FLAG overexpression impacted cell cycle progression. The 2D post-3D cells derived from T47D and MCF7 spheroids were synchronized by serum deprivation and analyzed for the effect of CCT2-FLAG overexpression on the cell cycle. Using PI staining for DNA analysis, we confirmed that about 70% of cells were in G1 after 24-hours of serum deprivation (**Figures S7A–D**). T47D CCT2 overexpressing cells had more cells in S and G2 phases after 24- and 48-hours post-serum addition compared to lentiviral control cells (**Figures 8C**, **S8C**). MCF7 CCT2 overexpressing and lentiviral control cells had comparable cell cycle distribution after serum addition, however these cells were inherently more proliferative (**Figures 8D**, **S8D**). Collectively, we found that CCT2 promotes breast cancer cell cycle progression through G1/S, in 2D, spheroid and 2D post 3D cultures; however, this effect could be cell type dependent and may involve different mechanisms depending on the cell’s genetic make-up.

### CCT2 Upregulates Expression of MYC and Cell Cycle Genes Under Different Culture Conditions

**Figure 9:**
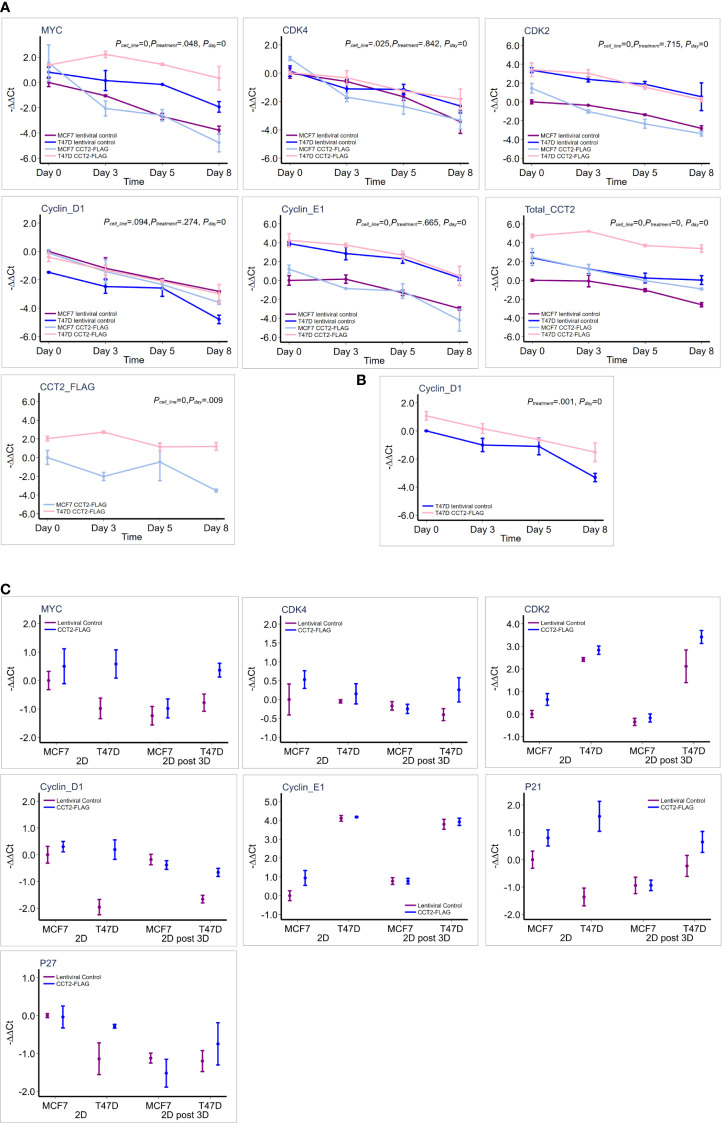
CCT2 upregulates expression of MYC and cell cycle genes in breast cancer cells. **(A)** Graphs show results of RT-qPCR for relative gene expression measured as (-ΔΔCt) for each gene in response to CCT2-FLAG overexpression in T47D and MCF7 cells at days 0 (pre-spheroid), 3, 5, and 8 of 3D spheroid cultures (n=3-5). The MCF7 lentiviral control was used as a reference sample. This data was used for the multiple linear mixed effect model shown in **Table 4**. **(B)** Cyclin D1 expression was evaluated in reference to T47D lentiviral control and statistical analysis shown in **Table 4**. **(C)** Comparison of gene expression is shown, measured as -ΔΔCt for each gene, in response to CCT-FLAG overexpression in T47D and MCF7 cells grown in 2D (pre-spheroid) (n=5) and 2D post 3D/spheroid (n=6) cultures. This data was used to perform the multiple factor ANOVA analysis shown in **Table 5**.

To further investigate how CCT2 promotes cell cycling, we determined whether increased levels of CCT2 correlated with changes in gene expression of key cell cycle regulators. The expression of MYC and others was assessed by RT-qPCR. GAPDH was used as a reference gene. Gene expression is presented as -ΔΔ Ct relative to the MCF7 lentiviral control reference sample, since these cells had the lowest CCT2 expression. MCF7 and T47D cell lines had inherent differences in expression of the tested genes (**Table 4**; **Figure 9A**). Collectively, T47D spheroids displayed statistically significant higher levels of MYC, CDK2, CDK4, and CCNE1 compared to MCF7 spheroids (**Table 4**). As spheroid cultures grew, expression for selected genes was downregulated, which is consistent with a general decrease in cellular processes observed with spheroid growth (53–55). Peaks of gene expression most commonly occurred on day 3 of spheroid culture, which correlated with peak levels of CCT2 (**Figure 9A**). Importantly, CCT2-FLAG overexpression significantly upregulated the expression of the cell cycle regulators, MYC (**Figure 9A**) and CCND1/cyclin D1 in T47D cells relative to the lentiviral control cells (**Figure 9B**, **Table 4**).

**Table 4:** Summary of multiple mixed effect linear regression analysis on expression of each gene in response to cell line (T47D vs MCF-7), treatment [lentiviral control (control) vs CCT2-FLAG overexpression (over-CCT2)] and day.

Gene	Factor	Estimated Coefficient	SE	P-value	Sig.
MYC	T47D vs. MCF7	*2.459*	*0.386*	*0*	***
	Over-CCT2 vs. Control	*0.765*	*0.386*	*0.048*	*
	Day	*-1.124*	*0.167*	*0*	***
	Intercept	-0.623	0.418	0.136	
CDK4	T47D vs. MCF7	0.525	0.235	0.025	*
	Over-CCT2 vs. Control	0.047	0.235	0.842	
	Day	-0.976	0.105	0	***
	Intercept	-0.062	0.257	0.81	
CDK2	T47D vs. MCF7	3.276	0.228	0	***
	Over-CCT2 vs. Control	-0.083	0.228	0.715	
	Day	-1.13	0.102	0	***
	Intercept	0.51	0.25	0.041	*
Cyclin_D1	T47D vs. MCF7	-0.579	0.348	0.096	
	Over-CCT2 vs. Control	0.381	0.348	0.274	
	Day	-0.981	0.069	0	***
	Intercept	-0.406	0.319	0.203	
Cyclin_D1 in T47D*	Over-CCT2 vs. Control	1.130	0.350	0.001	***
	Day	-0.930	0.101	0.000	***
	Intercept	0.037	0.290	0.899	
Cyclin_E1	T47D vs. MCF7	3.698	0.281	0	***
	Over-CCT2 vs. Control	0.122	0.281	0.665	
	Day	-1.266	0.126	0	***
	Intercept	0.696	0.308	0.024	*
Total_CCT2	T47D vs. MCF7	2.738	0.333	0	***
	Over-CCT2 vs. Control	2.448	0.333	0	***
	Day	-0.845	0.097	0	***
	Intercept	-0.089	0.323	0.784	
CCT2_FLAG	T47D vs. MCF7	3.276	0.565	0	***
	Over-CCT2 vs. Control				
	Day	-0.657	0.253	0.009	**
	Intercept	-0.522	0.551	0.344	

*MCF-7 lentiviral control was used as the reference samples for all analysis except for cyclin D1 in T47D cells in which the T47D lentiviral control was used as the reference sample.

Day is set as a continuous variable with value of 0 for day 0, 1 for day 3, 2 for day 5 and 3 for day 8 of spheroid culture. *p <0.05, **p<0.005, ***p<0.0005, SE, standard error.

Next, two different populations of cells grown in 2D monolayer cultures were evaluated, the more heterogeneous cells that were adapted to 2D adherent growth conditions and the 2D post 3D cells, that underwent spheroid growth reversal. CCT2-FLAG overexpression upregulated the expression of MYC, CCND1, CDK2, and CDKN1A (**Table 5**). Especially significant was the increased expression of MYC and CCND1 in T47D CCT2-FLAG overexpressing cells as compared to lentiviral controls (**Figure 9C**, **Table 5**). In summary, we found that in CCT2 overexpressing cells (especially T47D cells) gene expression of key regulators of cell proliferation, such as MYC and CCND1, was increased and potentially contributed to the proliferative expansion induced by CCT2 detected across 2D, 3D and 2D post-3D culture conditions.

**Table 5:** Summary of multi-factor ANOVA analysis showing effect of individual factors on the expression of each gene in response to effects of cell line (T47D vs MCF-7), treatment [lentiviral control (control) vs CCT2-FLAG overexpression (over-CCT2)] and 2D (before spheroid culture) vs 2D post 3D (after spheroid growth reversal).

Gene	Factor	Estimated Coefficient	SE	P-value	Sig.
MYC	T47D vs. MCF7	0.455	0.279	0.113	
	Over-CCT2 vs. Control	0.815	0.279	0.007	**
	2D post 3D vs. 2D	-0.69	0.301	0.029	*
	Intercept	-0.603	0.308	0.059	
CDK4	T47D vs. MCF7	0.019	0.163	0.906	
	Over-CCT2 vs. Control	0.314	0.163	0.063	
	2D post 3D vs. 2D	-0.3	0.173	0.093	
	Intercept	-0.009	0.182	0.961	
CDK2	T47D vs. MCF7	2.784	0.287	0	***
	Over-CCT2 vs. Control	0.667	0.287	0.027	*
	2D post 3D vs. 2D	-0.221	0.305	0.474	
	Intercept	-0.253	0.321	0.436	
Cyclin_D1	T47D vs. MCF7	-0.977	0.197	0	***
	Over-CCT2 vs. Control	0.63	0.197	0.003	**
	2D post 3D vs. 2D	-0.289	0.212	0.182	
	Intercept	-0.259	0.217	0.242	
Cyclin_E1	T47D vs. MCF7	3.275	0.172	0	***
	Over-CCT2 vs. Control	0.201	0.172	0.251	
	2D post 3D vs. 2D	0.022	0.185	0.905	
	Intercept	0.559	0.19	0.006	**
P21	T47D vs. MCF7	0.377	0.368	0.319	
	Over-CCT2 vs. Control	1.101	0.368	0.008	**
	2D post 3D vs. 2D	-0.565	0.368	0.142	
	Intercept	-0.54	0.359	0.149	
P27	T47D vs. MCF7	-0.179	0.268	0.514	
	Over-CCT2 vs. Control	0.213	0.268	0.436	
	2D post 3D vs. 2D	-0.777	0.268	0.009	**
	Intercept	-0.388	0.262	0.155	

*p <0.05, **p<0.005. ***p<0.0005. SE, standard error.

### CCT2 as a Possible Oncogene

**Figure 10:**
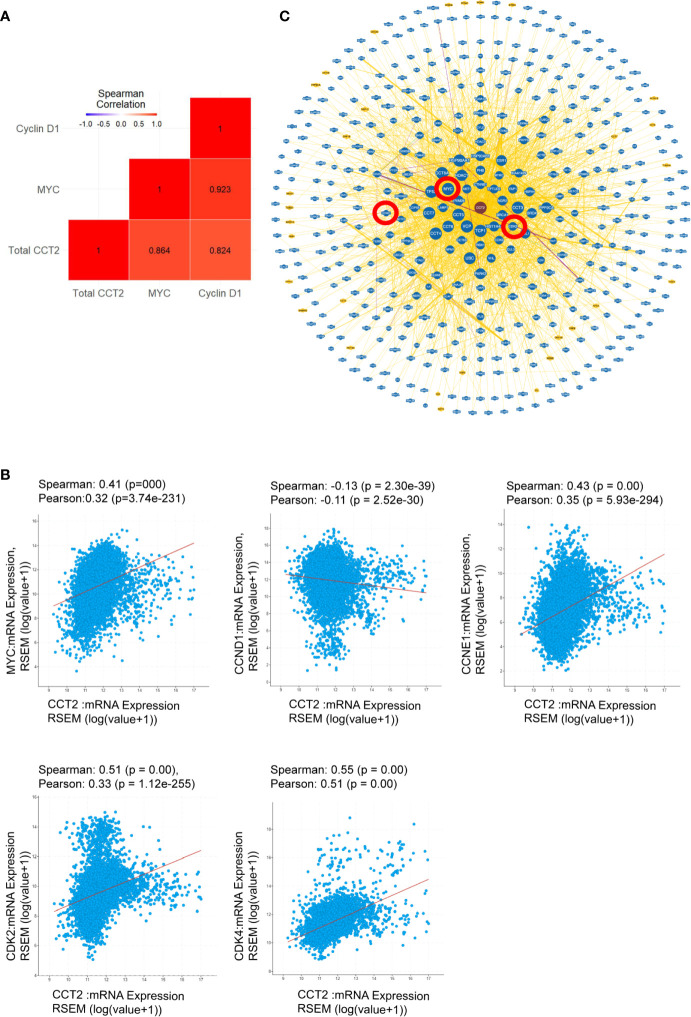
CCT2 as a possible oncogene. **(A)** Spearman correlation coefficients were determined to demonstrate gene interactions among MYC, CCND1 (Cyclin D1), and total CCT2 in MCF7 and T47D cells, CCT2 overexpressing and lentiviral controls. Results from the combined dataset are shown (n=3). Gene expression was measured as -ΔΔCt using the MCF7 lentiviral control as reference. The red colored squares indicate that correlations were significant with p-values <0.05. P-values were adjusted based on the Holm multiple test adjustment. **(B)** The TCGA PanCancer dataset was analyzed for co-expression of CCT2 mRNA with MYC, CCND1, CCNE1, CDK2, and CDK4 mRNA. Spearman and Pearson correlation p-values are shown. **(C)** The CCT2 interaction network with physical and genetic integrators is shown (data from BioGRID). The positions of MYC, CCND1,and CDK2 in the CCT2 interactome are highlighted in red circles. Greater node size represents increased connectivity and thicker edge sizes represent increased evidence supporting the association. The list of CCT2 interactors is included in **Supplementary Data** (see Excel sheet).

The highest gene expression for MYC in the CCT2-FLAG overexpressing T47D cell line was observed on day 3 of spheroid culture (**Figure 9A**) and is consistent with total CCT2 mRNA expression (**Figure 9A**). These results led us to determine whether there was a statistically significant correlation between CCT2, MYC, and CCND1 in the breast cancer cell lines studied. Gene expression of CCT2, MYC and CCND1 was assessed from the same batch of 2D and day 3 spheroid cultures of T47D and MCF7 cells, CCT2-FLAG and lentiviral control. Combining the datasets to increase statistical power, we performed a gene correlation analysis and found that total CCT2 expression significantly correlated with the increased expression of MYC and CCND1, with Spearman correlations of 0.864 and 0.824, respectively, and p< 0.05 (**Figure 10A**). To determine if the correlation between CCT2, MYC, and CCND1 was clinically relevant, we analyzed TCGA data for CCT2 mRNA co-expression with these genes. Using combined pan-cancer studies, moderately positive correlations of CCT2 mRNA were found with MYC, CDK2, CDK4, CCNE1 but not CCND1 (slight negative correlation) (**Figure 10B**). However, a different correlation test using the UCSC Xena database (study: TCGA BRCA) did find a weak but significant correlation between CCT2 and CCND1 (Pearson’s correlation 0.095, p-value < 0.001). These findings suggest that CCT2 could be a possible node for the interaction of key cell cycle regulators in the uncontrolled growth of cancer cells. The scope of the proliferative factors interacting with CCT2 is shown in an analysis of the network resulting from compiling data of potential CCT2 interactors (evidence from both physical and genetic interactions, BioGRID). MYC, CDK2, and cyclin D1 (highlighted by red circles) as part of the CCT2 interactome are shown, supporting that CCT2 could be a central point or node for regulation of cancer proliferation pathways mediated by these factors (**Figure 10C**). The full list of interactors is shown in **Supplementary Data** (Excel sheet).

CCT2 is located on chromosome 12q15 along with other identified oncogenes MDM2, FRS2, YEATS4 and others. CDK4 (12q13) also spans the chromosomal region 12q13-15. Hence CCT2 is part of an amplicon that is associated with cancer development. The highest percent of samples/patients with CCT2 gene amplification or increased gene expression is observed in soft tissue cancers like sarcoma (19% of sarcoma samples/patients have gene alterations in CCT2, TCGA) (**Figure S9A**). CCT2 is also genetically altered in invasive breast cancer along with other 12q15 oncogenes, the percent of which varies depending on the study and availability of samples (**Figures S9B, C**). Along with its role in the CCT protein folding complex promoting the folding of substrates like actin, we showed that CCT2 supports the proliferation of cancer cells in 2D and 3D conditions, correlates with the expression of oncogenes like MYC, and thus may fulfill the basic requirements for an oncogene as it undergoes genomic amplification during the mutational processes that drive tumorigenesis (56, 57).

## Discussion

The CCT complex assists in the folding, stability, maturation, or assembly of many proteins essential for cancer cells. However, being a large complex composed of eight subunits, the therapeutic targeting of CCT is challenging. To address this, we show that overexpressing one subunit, CCT2, is sufficient to promote the proliferation of cancer cells and enhance the potential for metastasis, suggesting that this subunit could be targeted to inhibit the carcinogenic activity of the complex. We chose two luminal A breast cancer cell lines that had different genetic backgrounds and low endogenous levels of CCT2 (40, 42) to study the function of CCT2. T47D cells, which are also PR high (58), manifested the greatest changes upon CCT2 overexpression, with increased spheroid size and numbers and enhanced proliferation. T47D cells also sustained higher levels of CCT2-FLAG expression, displayed spheroid growth reversal, and anchorage-independent growth. Importantly, increases in the gene expression of MYC and CCND1 correlated with CCT2 in T47D cells, suggestive of a possible mechanism driving the increased cell cycling. The data from T47D cells show that overexpressing CCT2 can result in the uncontrolled proliferation and the metastatic-like behavior of aggressive, invasive cancer cells and is linked to increased expression of cell cycle regulators. In contrast, MCF7 cells, which have higher CCT2 copy number compared to T47D cells, inherently do not express more CCT2 but rather have high endogenous levels of MYC and CCND1. While CCT2-FLAG overexpression was achieved in these cells, high levels of total CCT2 were not sustained. As a result, we did not observe significant increases in spheroid growth or spheroid growth reversal over that of MCF7 control cells. Taken together, the data from these cell lines support that CCT2 is a novel regulator of cell proliferation that could be a node for the interaction of growth pathways involving cell cycle regulators like MYC and CCND1.

The pro-growth activity of CCT2 can be in part explained through the protein-folding activity of the chaperonin complex. Increasing CCT2 could in turn increase the folding and availability of functional forms of proteins involved in cell cycling like cyclin E (37) and cdc20 (35) that are substrates of the chaperonin. In support, one study showed that CCT2 is essential for the allosteric cooperativity of the chaperonin, increasing complex functionality (59). The CCT2 interactome is not fully known, but information from databases like BioGrid indicates that CCT2 may have physical and genetic interactions with more than 500 proteins, many of which like MYC and CCND1 drive cell cycling and promote carcinogenesis. Our data also suggests that additional mechanisms beyond protein-folding and biogenesis may directly influence the gene expression of key cell cycle regulators like CCND1 and CDK2 and growth promoters like MYC. One way could be by acting as an RNA binding protein (RBP), since CCT2 was shown to interact with MYC mRNA and could govern the biology of this mRNA (60). How CCT2 increases the expression of CCND1 or CDK2 remains unknown but could be an indirect result of increasing MYC or other transcription factors.

MYC is a major proto-oncogene in breast cancer, especially for HER2 and BRCA-1 associated cancers that have poor prognoses (19, 61, 62). Likewise, CCND1 (cyclin D1) is amplified or overexpressed in cancer and may cooperate with MYC to promote transformation (63). Herein, we showed that CCT2 expression can upregulate MYC and CCND1 gene expression and promote metastatic-like behavior in luminal A breast cancer cells that are typically less aggressive than the basal-like/TNBC subtypes usually associated with MYC amplification. Others reported that overexpression or suppression of CCT3 in basal-like TNBC cells altered cell proliferation and changed the expression of MYC (64, 65). One pathway of interest is WNT/β-catenin signaling that regulates cell growth and could drive proliferation in breast cancer cells through MYC and CCND1 (66, 67). Overexpression of CCT3 increased β-catenin in MDA-MB-231 and T47D breast cancer cells, which could be modulated by microRNA (miRNA) 223. This miRNA was shown to bind to the 3′UTR of both CCT3 and β-catenin (65). The conclusion drawn from this study was that CCT3 mediates breast cancer growth by binding to and competitively inhibiting miRNA 223. However, a prediction analysis for miRNA targets in CCT2 did not reveal similar findings (http://www.targetscan.org/cgi-bin/targetscan/vert_72/view_gene.cgi?rs=ENST00000299300.6&taxid=9606&members=&showcnc=0&shownc=0&showncf1=&showncf2=&subset=1). Moreover, we noted that CCT3 mRNA and protein in our study did not vary substantially between the lentiviral control and CCT2-FLAG overexpressing breast cancer cells. While we cannot definitively rule out the contribution of miRNAs and other CCT subunits in the effects of CCT2 overexpression upon MYC, cyclin D1 and subsequent proliferation and spheroid growth, the mechanisms driving this interaction remain to be elucidated. One possible avenue for further exploration is the relationship between CCT2, MYC and p53. In a study of mutant TP53 in head and neck cancers, CCT2 was identified as a MYC and mutant TP53 target gene. Depletion of mutant p53 reduced the interaction of MYC with the CCT2 promoter (68). Since T47D cells have a missense TP53 mutation with potential gain of function (MCF7 have wild type p53), it is interesting to speculate that the mutant TP53-MYC axis may be involved in the responsiveness of this cell line to CCT2 overexpression.

Using 3D cultures to model breast tumor growth we observed increased spheroid formation upon CCT2 over expression, as evidenced by multiple and larger spheroids, and loss of tight cell contacts that support spheroid structure when CCT2 was depleted. In support, we also found that actin was upregulated in CCT2 overexpressing cells, especially as cells transitioned from 3D to monolayer cultures. One group showed that cells undergoing 3D to 2D culture transitions retained signatures typical of metastatic cells, such as the expression of cancer stem-cell markers and the development of chemoresistance (51). Our data suggest that CCT2 could enhance the potential of spheroids to not only grow but to transition to 2D culture, which is suggestive of a more aggressive phenotype, and such a phenotype that could be responsible for promoting drug resistance. For example, spheroids from MDA-MB-231 cells, which we previously reported had high endogenous levels of CCT2 (42), developed increased resistance to treatment with carboplatin or doxorubicin (69).

The gene for CCT2 is located in the 12q15 amplicon, which also contains the oncogenes MDM2, YEATS4, FRS2 among others. High level amplification of this amplicon may be an early event in precursor cells that give rise to sarcomas (70) and other cancers like gliomas and melanomas (71). Genomic imbalances in this region are also found in other cancers such as follicular lymphomas (72). Given this, therapeutically targeting CCT is complicated by the fact that this is a multi-subunit complex, which challenges the development of small molecule inhibitors. Others identified a small molecule that interferes with the CCT2-β−tubulin interaction that could have application in the treatment of resistance to tubulin-binding agents (73). However, given the genetic heterogeneity of cancer, a CCT inhibitor that works independently of substrate identification is needed. To this end, our lab previously identified a small amphipathic peptide, called CT20p, as being cytotoxic in cancer cells that highly express CCT (42, 43). Using polymeric nanoparticles to systemically deliver CT20p, we achieved regression of breast and prostate tumors in mice (74–76) and demonstrated that CT20p directly binds to the CCT2 subunit (42). CT20p thus demonstrates that CCT2 and the chaperonin complex can be therapeutically targeted and have biological outcomes such decreasing tumor growth. Herein we show the value of CCT2 as a druggable target by demonstrating its role as a cell cycle regulator that intersects with key proliferative factors like MYC and CCND1. Future studies on establishing the function of CCT2 as an oncogene and elucidating its relationship with MYC could undercover a novel mechanism underlying cancer growth and dissemination and reveal uses for CCT inhibitors in the treatment of drug resistant cancers and in the prevention of cancer relapse and metastasis. 

## Data Availability Statement

The original contributions presented in the study are included in the article/**Supplementary Material**, further inquiries can be directed to the corresponding author.

## Author Contributions

The manuscript was written through contributions of all authors. HG, AS, EL, XZ, and AK. HG and AS contributed to data acquisition. EL and XZ contributed to data analysis. HG, AS, and AK contributed to the experimental design and manuscript preparation. All authors contributed to the article and approved the submitted version. 

## Funding

Funding was provided by the Breast Cancer Research Foundation (BCRF) (BCRF-18-086).

## Conflict of Interest

AK is a shareholder in Seva Therapeutics, Inc.

The remaining authors declare that the research was conducted in the absence of any commercial or financial relationships that could be construed as a potential conflict of interest.

## Supplementary Material

Supplementary Figure 1 CCT3 expression in breast cancer CCT2-FLAG overexpressing cells. **(A)** Representative immunoblots of CCT3 protein expression in T47D and MCF7, CCT2-FLAG overexpressing and lentiviral control, cells are shown. Graph summarizes data from blots that was normalized to total protein. **(B)** CCT3 relative mRNA expression was assessed using RT-qPCR. GAPDH was used as the reference gene.

Supplementary Figure 2 CCT2 protein levels in spheroid cultures of breast cancer cells. Graphs show total CCT2 protein, CCT2-FLAG protein (anti-FLAG) and endogenous CCT2 protein normalized to total protein in T47D **(A)** and MCF7 **(B)**, CCT2-FLAG overexpressing and lentiviral control, cells. Data was determined from immunoblots shown in **Figure S3**.

Supplementary Figure 3 Representative immunoblots for CCT2 total protein expression in T47D **(A)** and MCF7 **(B)**, CCT2-FLAG (anti-FLAG) and endogenous CCT2 in T47D **(C)** and MCF7 **(D)** at day 0 (2D cultures, pre-spheroid), spheroid cultures on days 3, 5, and 8, and 2D post 3D cultures are shown. Total protein stain is showed below each panel.

Supplementary Figure 4 CCT2 supports anchorage independent growth in culture. Dissociated spheroids from T47D lentiviral control **(A)** and CCT2-FLAG expressing cells **(B)** were grown in standard tissue culture flasks. Representative brightfield images at 20X magnification from various stages of growth were acquired after 3 weeks post transfer from spheroid culture. CCT2-FLAG overexpressing cells formed 3D spheroid like structure in monolayer culture conditions **(B)**, while lentiviral control cells grew in 2D cell sheets confined by the well **(A)**.

Supplementary Figure 5 CCT2 protein levels in spheroid reversal cultures (2D post 3D) of breast cancer cells. Graphs show **(A)** CCT2-FLAG (anti-FLAG), **(B)** total CCT2 protein, and **(C)** endogenous CCT2 protein expression normalized to total protein in T47D and MCF7, CCT2-FLAG overexpressing and lentiviral control, cells. Cells were grown in 3D spheroid cultures and then transferred to standard tissue culture plates for 2D growth (2D post 3D). Data was determined from immunoblots shown in **Figure S3**.

Supplementary Figure 6 Viability gating of breast cancer cells in spheroid culture. T47D and MCF7, CCT2-FLAG overexpressing and lentiviral control, cells were cultured on ULA plates and cells recovered for PI exclusion staining. Live cell gating on PI negative cells on days 3, 5, 8 of spheroid growth is shown for T47D **(A)** and MCF7 **(B)** cells.

Supplementary Figure 7 Serum deprivation causes cell cycle arrest of breast cancer cells. T47D and MCF7, CCT2-FLAG overexpressing and lentiviral control, cells were deprived of sera for 24 hours, and cells were collected for analysis of cell cycle distribution using PI staining. **(A–D)** Cell cycle distribution of T47D and MCF7 cells in standard 2D culture **(A, B)** and of cells transitioning from 3D to 2D culture (2D post 3D culture) **(C, D)** is shown.

Supplementary Figure 8 Cell Cycle distribution of growth synchronized cultures of breast cancer cells. Growth arrested T47D and MCF7, CCT2-FLAG overexpressing and lentiviral control, cells were cultured in sera-containing media for 24 to 48 hours, and cells were collected for analysis of cell cycle distribution using PI staining. **(A–D)** Cell cycle distribution of T47D and MCF7 cells in standard 2D culture **(A, B)** and of cells transitioning from 3D to 2D culture (2D post 3D culture) **(C, D)** is shown.

Supplementary Figure 9 CCT2 is located in an amplicon associated with cancer progression. CCT2 is found in 12q13-15 chromosomal region that is subject to recurrent amplification in cancer. **(A–C)** CCT2, MDM2, FRS2, YEATS4, CDK4 genetic alteration and heatmap for mRNA expression is shown from TCGA cases PanCancer combined and sarcoma **(A)** and invasive breast carcinoma **(B)**. Co-amplification of genes in the CCT2 altered group compared to unaltered group in invasive breast carcinoma (TCGA PanCancer studies) is shown **(C)**.
