# Supplementary material for: Chaperonin-Containing TCP1 Complex (CCT) Promotes Breast Cancer Growth Through Correlations With Key Cell Cycle Regulators
Source: Front Oncol. 2021 Apr 30;11:663877. doi: 10.3389/fonc.2021.663877 (PMC8121004; doi:10.3389/fonc.2021.663877)

Supplemental Figure 1

A

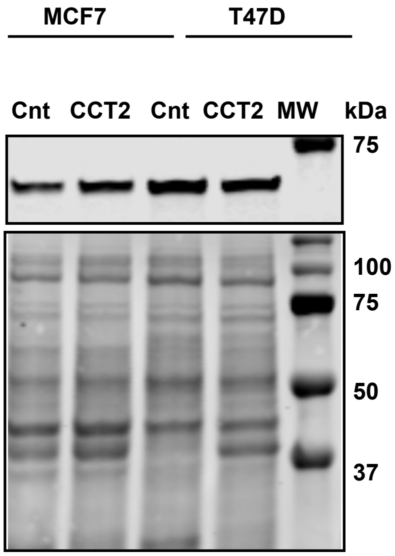

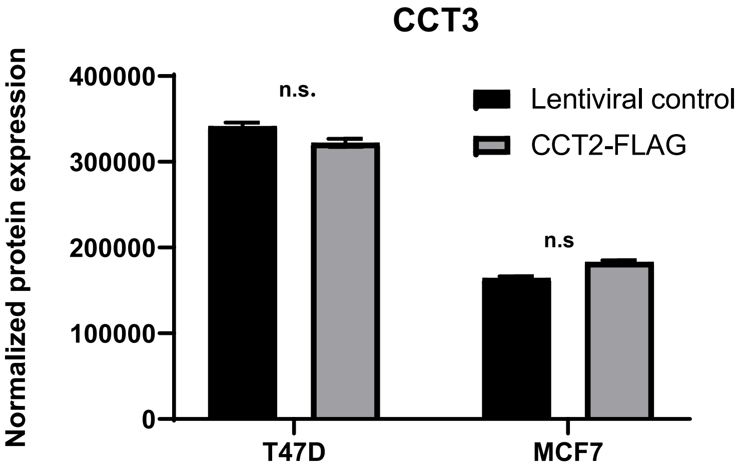

B

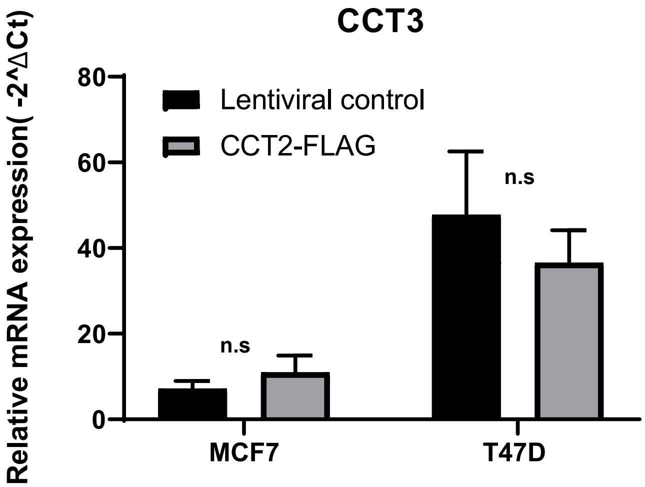

Supplemental Figure 2

A

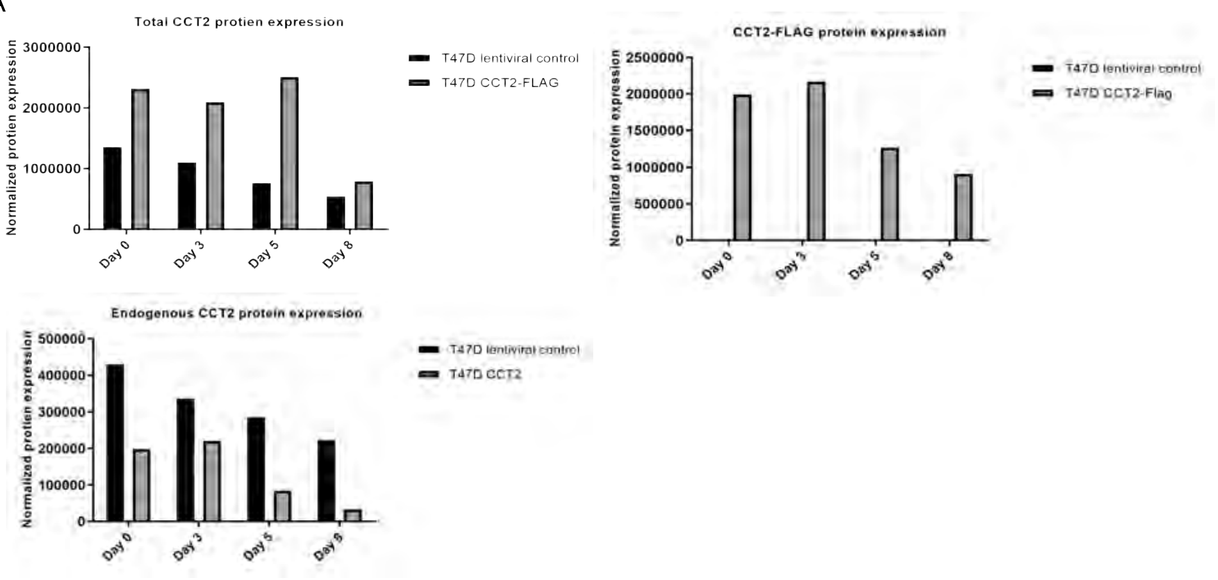

B

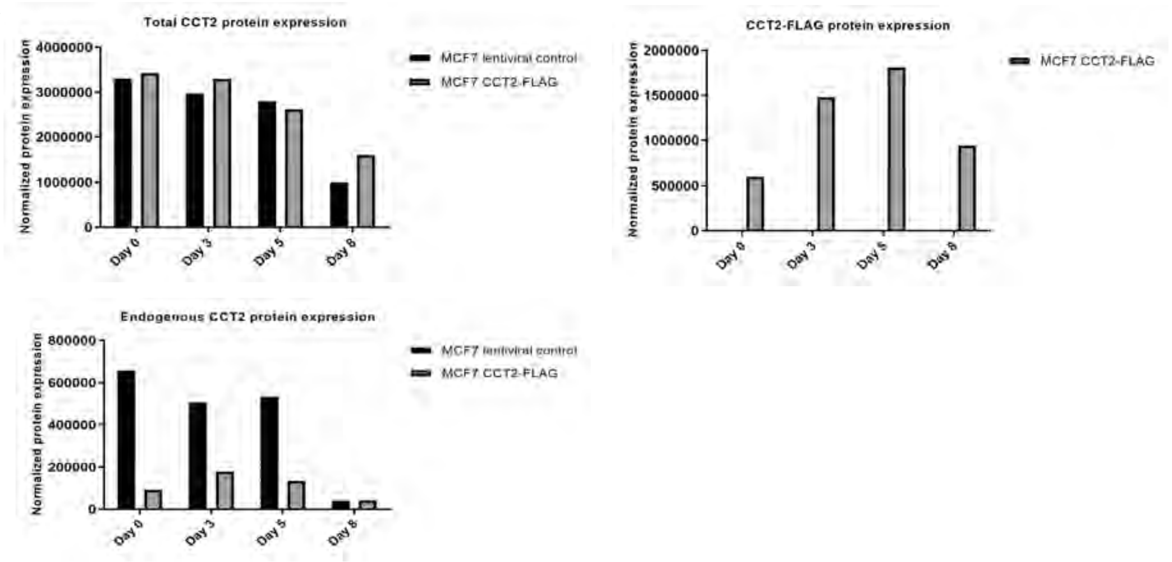

Supplemental Figure 3

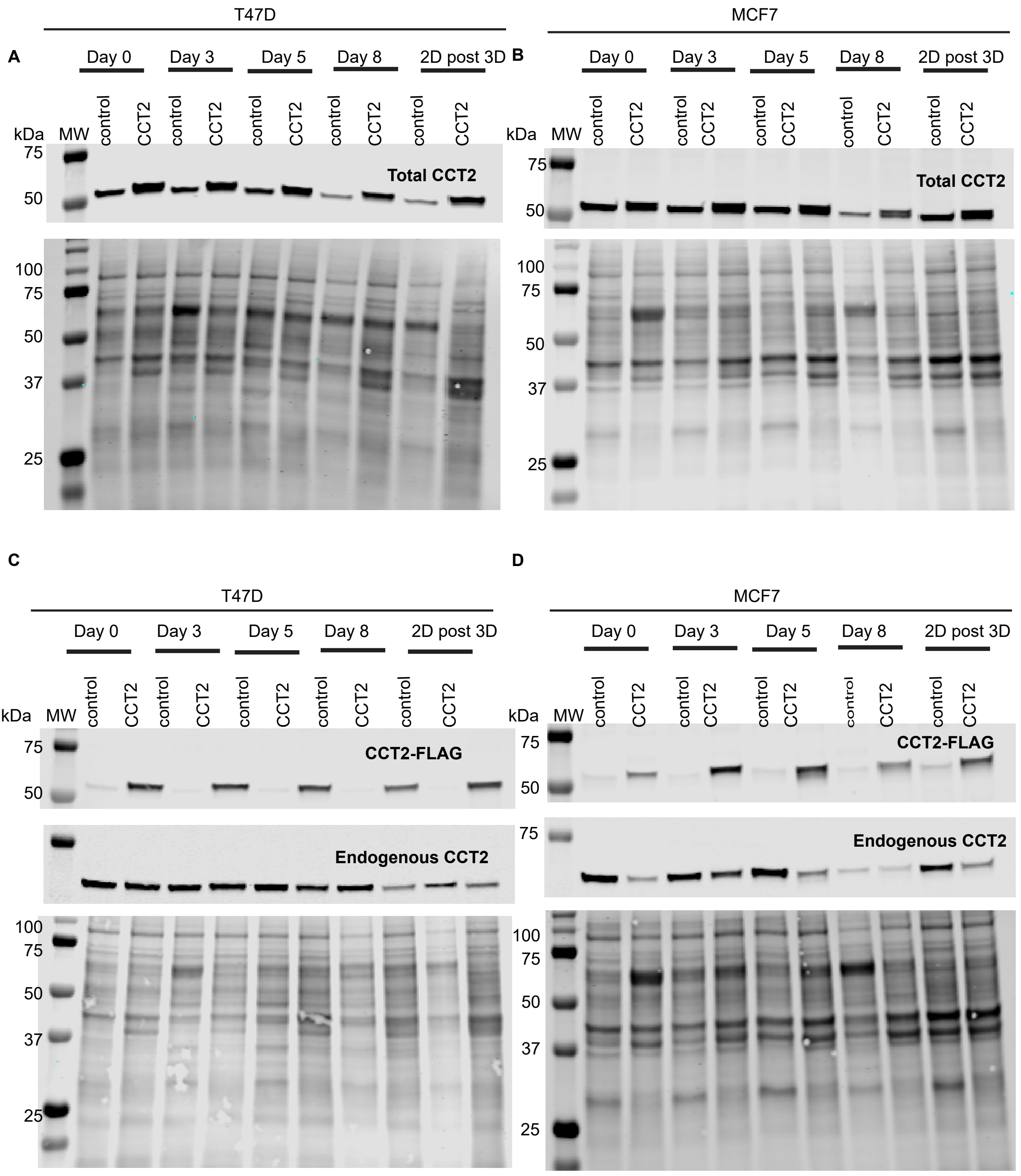

Supplemental Figure 4

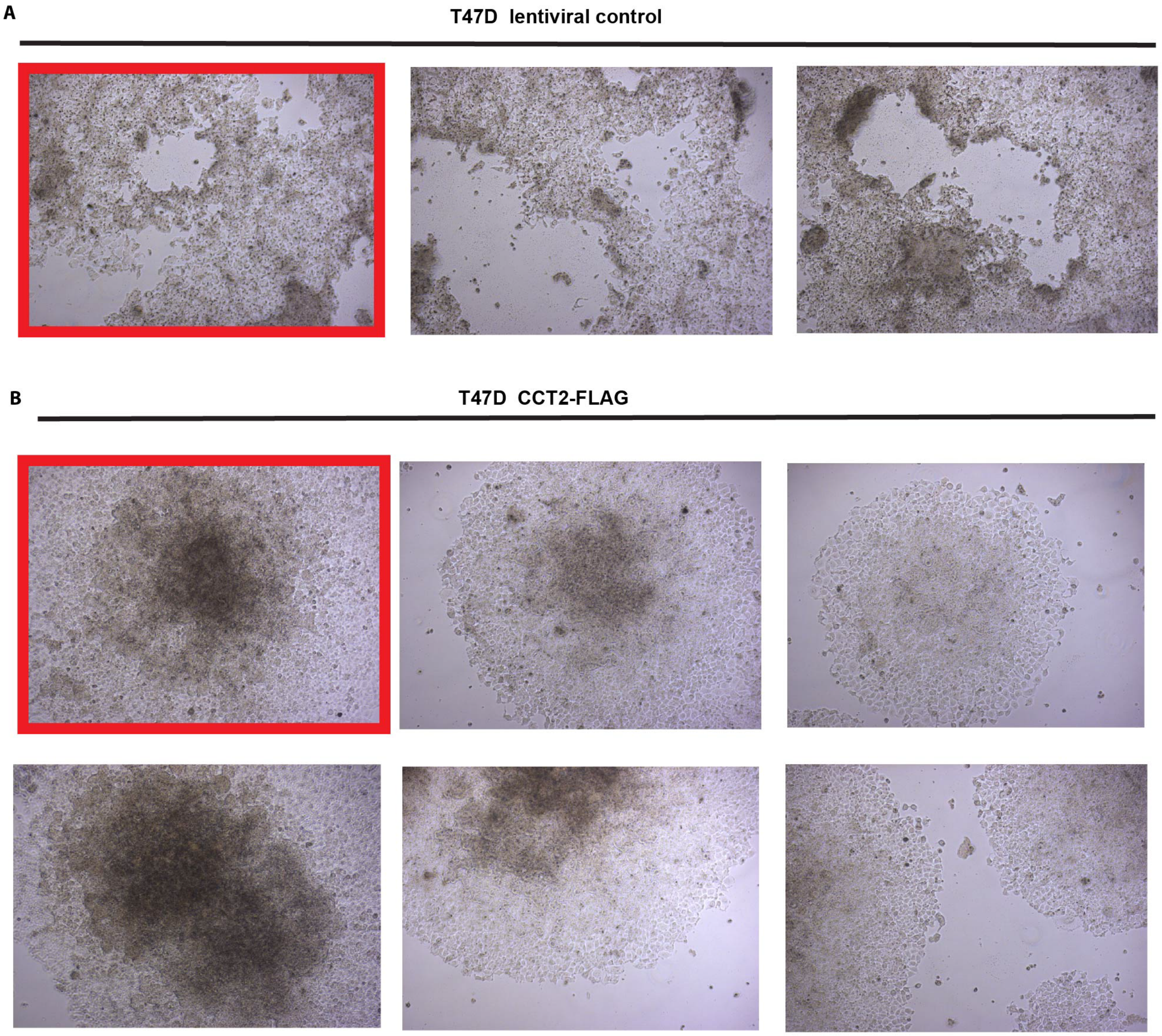

Supplemental Figure 5

A

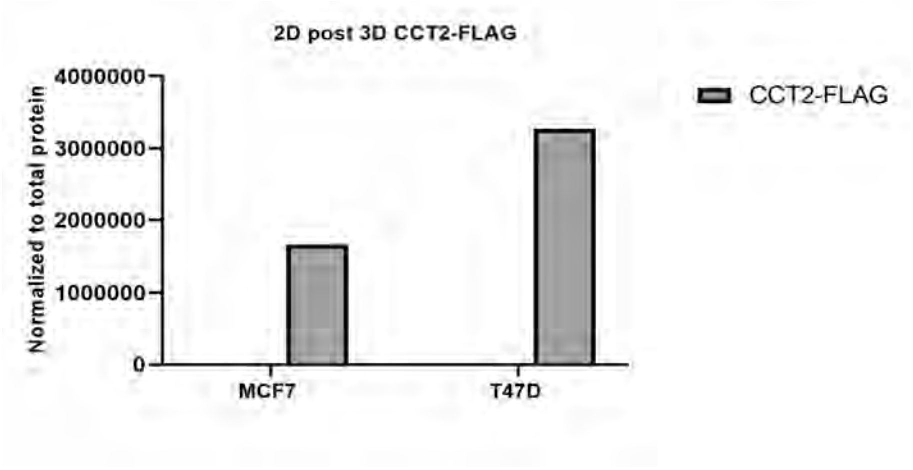

B

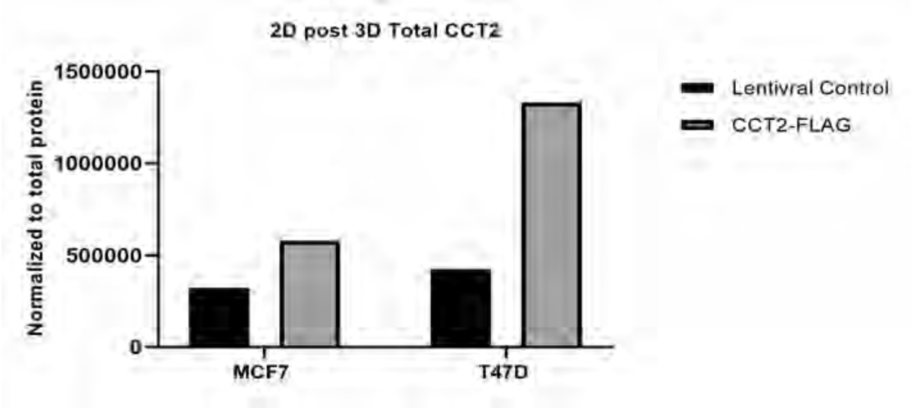

C

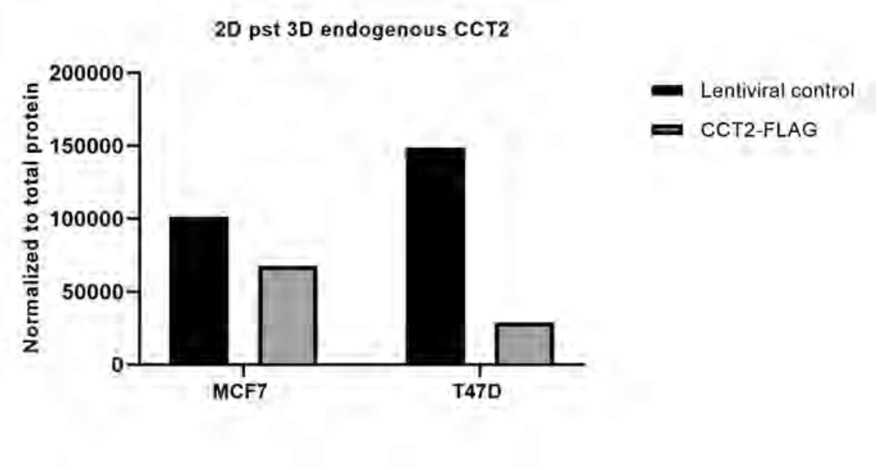

Supplemental Figure 6

A

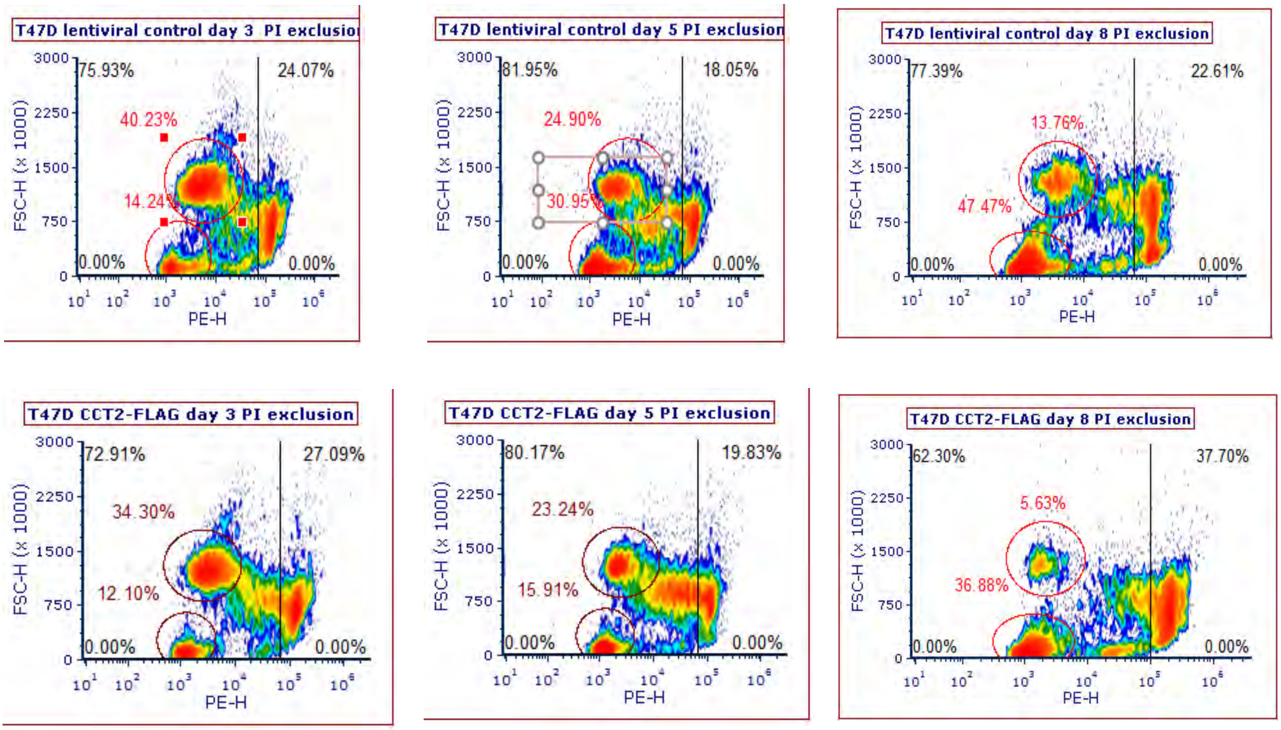

B

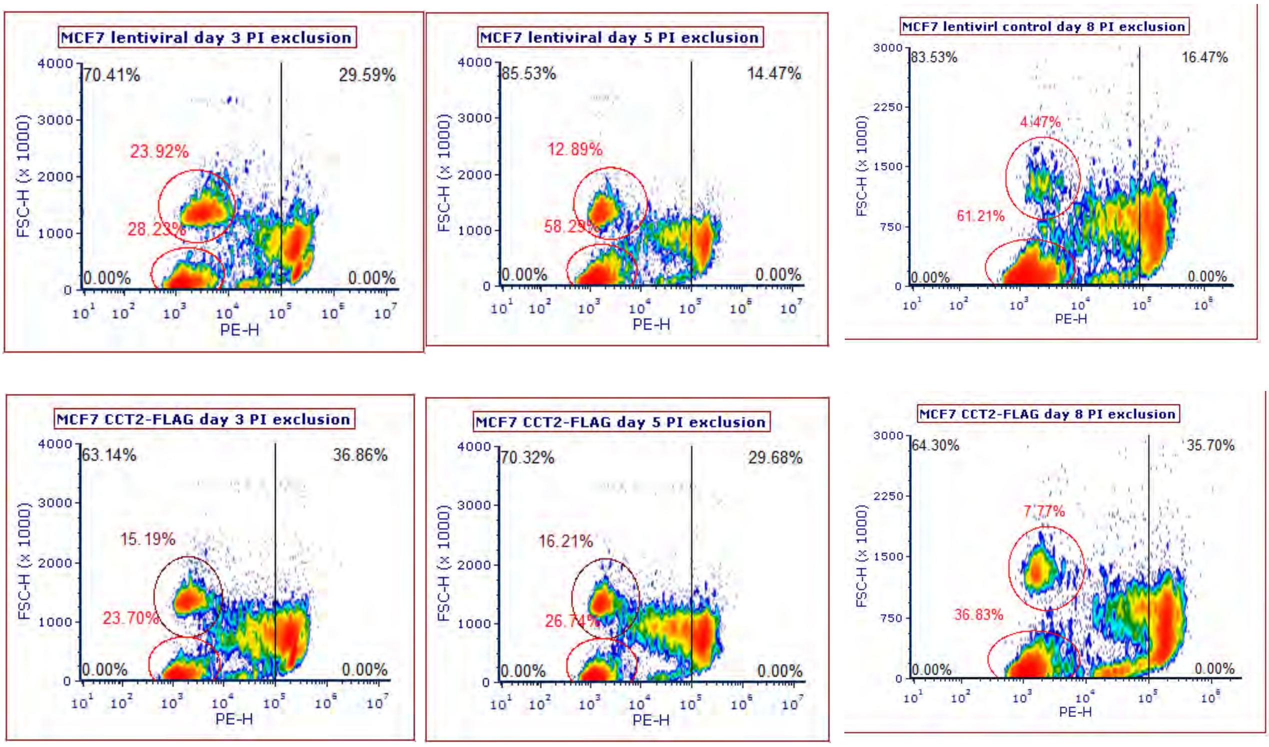

Supplemental Figure 7

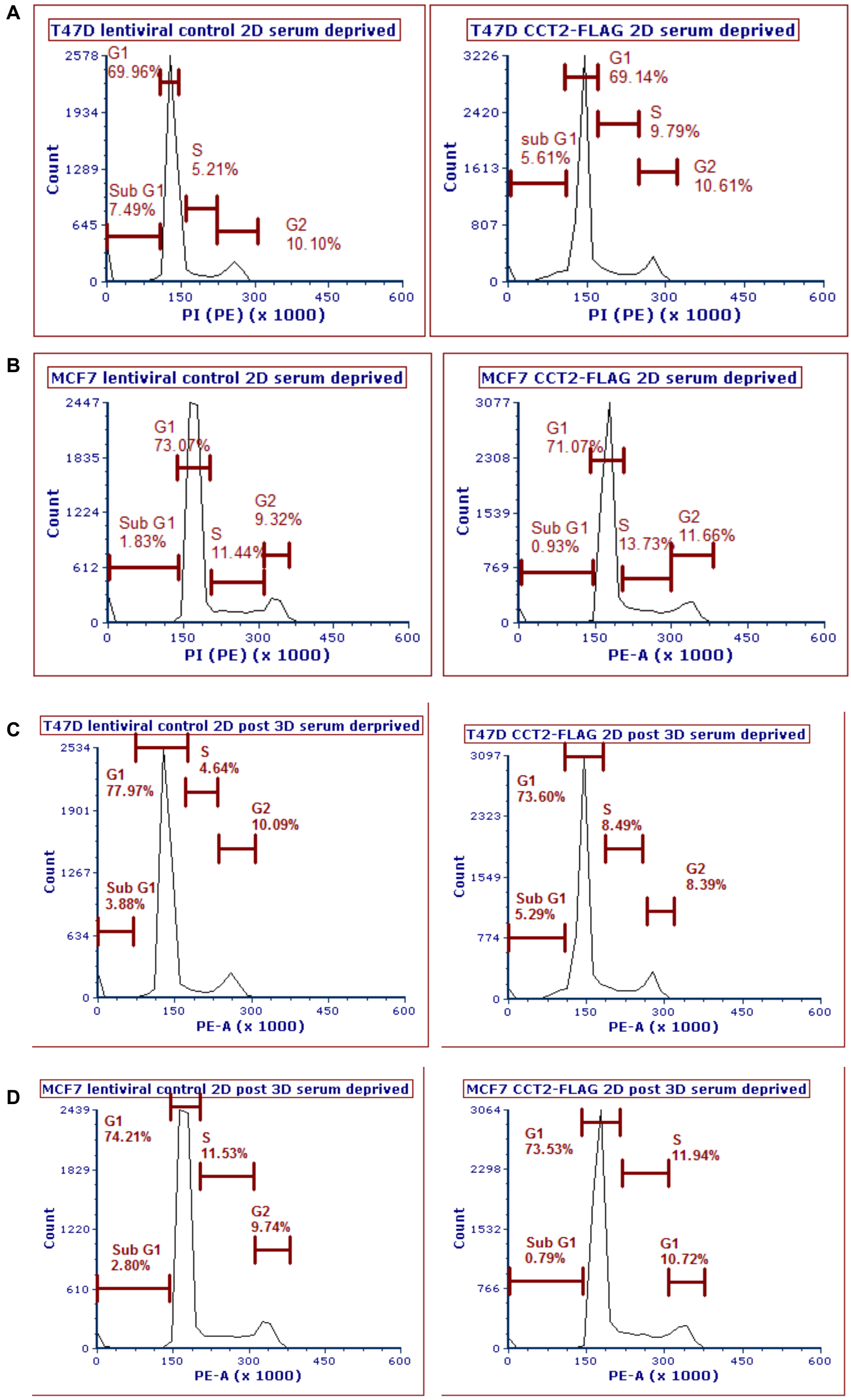

Supplemental Figure 8

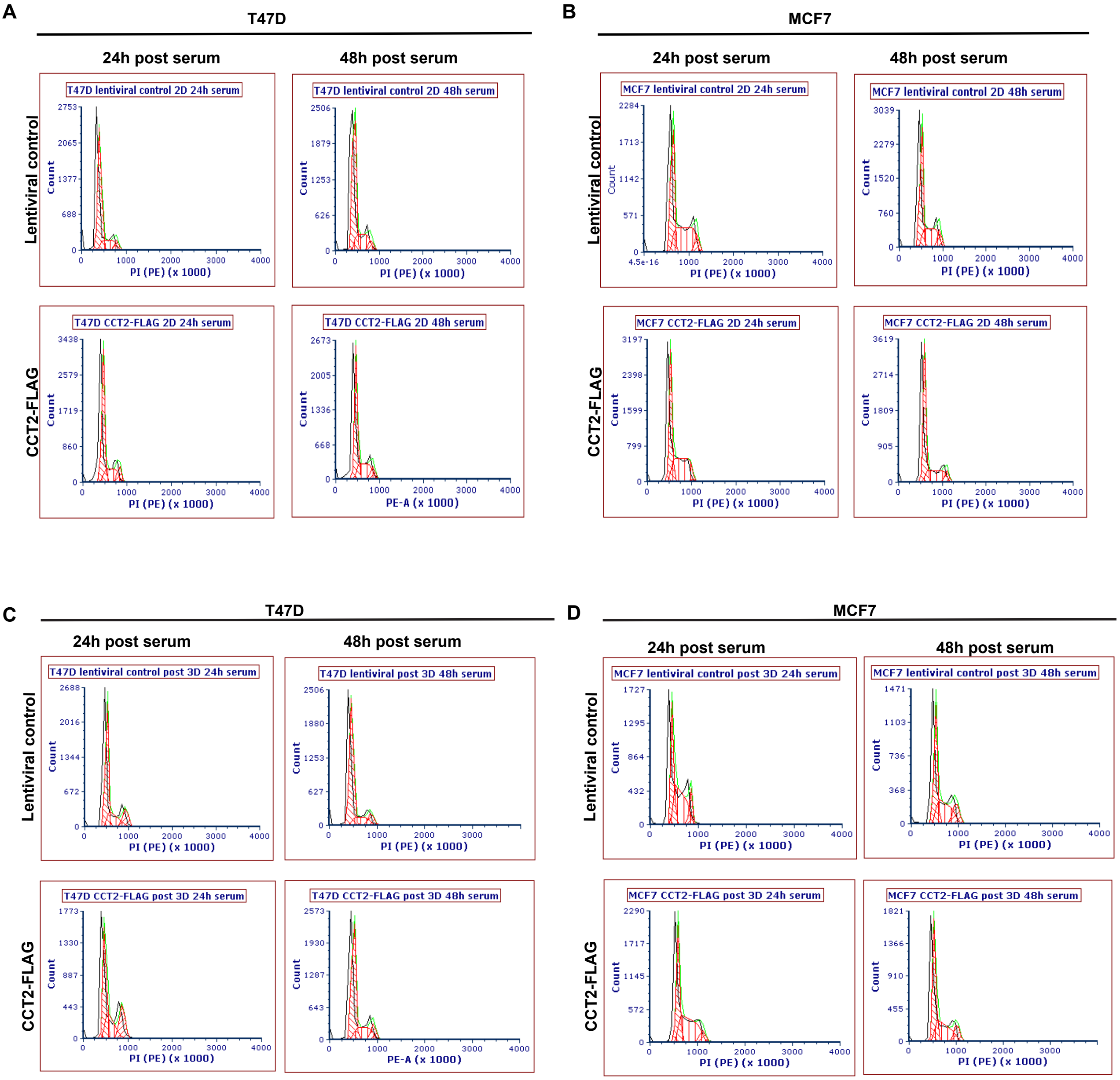

Supplemental Figure 9

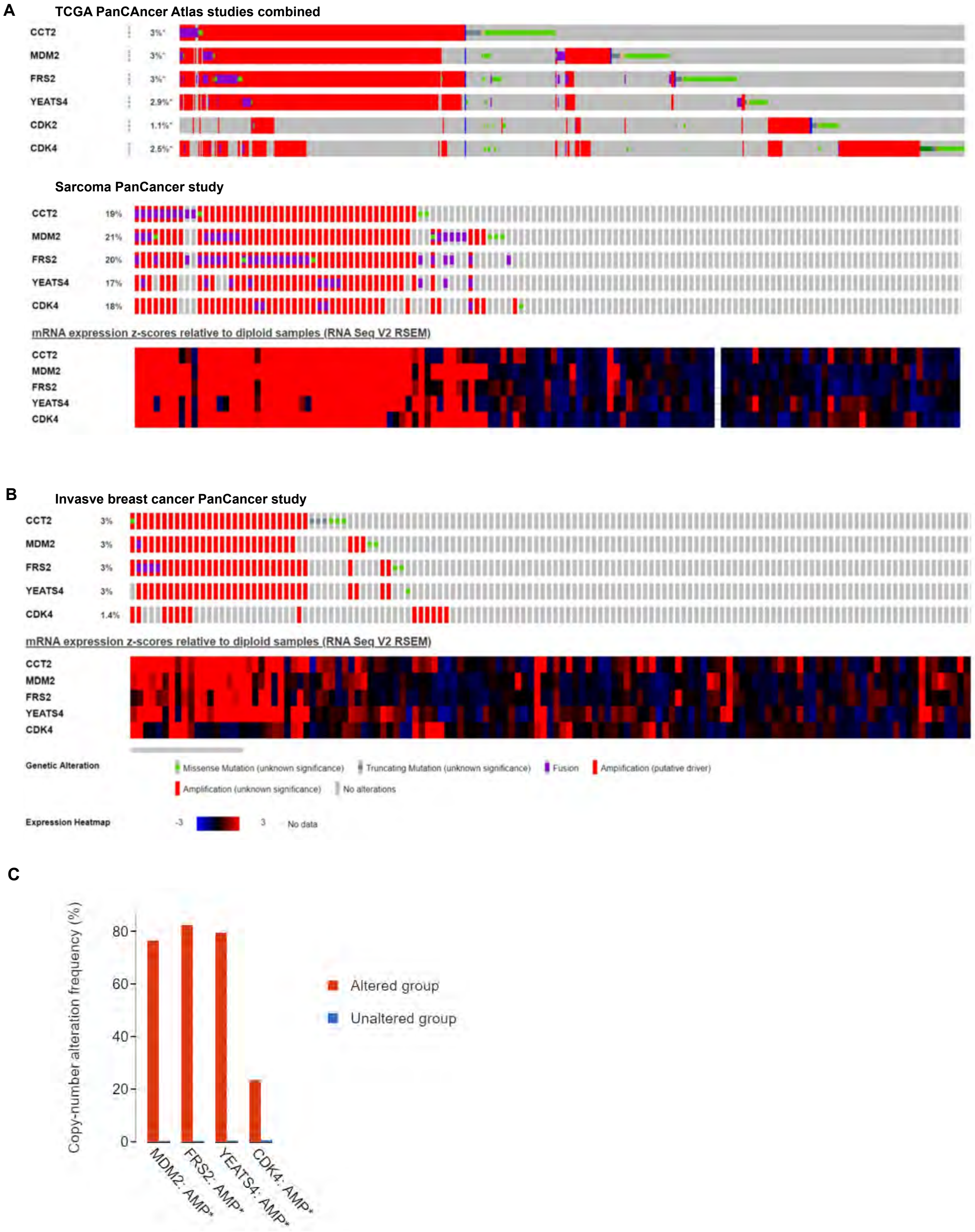

Supplement: Supplementary Figure 1 — CCT3 expression in breast cancer CCT2-FLAG overexpressing cells. (A) Representative immunoblots of CCT3 protein expression in T47D and MCF7, CCT2-FLAG overexpressing and lentiviral control, cells are shown. Graph summarizes data from blots that was normalized to total protein. (B) CCT3 relative mRNA expression was assessed using RT-qPCR. GAPDH was used as the reference gene. [file DataSheet_1.pdf]
